# UNC‐120/SRF independently controls muscle aging and lifespan in *Caenorhabditis elegans*

**DOI:** 10.1111/acel.12713

**Published:** 2018-01-03

**Authors:** Adeline Mergoud dit Lamarche, Laurent Molin, Laura Pierson, Marie‐Christine Mariol, Jean‐Louis Bessereau, Kathrin Gieseler, Florence Solari

**Affiliations:** ^1^ University of Lyon University of Lyon1 Claude Bernard Lyon1 NeuroMyoGene Institute CNRS UMR5310 INSERM U1217 Lyon France; ^2^ Hospices Civils de Lyon Faculté de Médecine Lyon Est Lyon France

**Keywords:** biomarkers, *Caenorhabditis elegans*, DAF‐2/insulin‐IGF‐1 receptor, muscle, transcription factor, UNC‐120/SRF

## Abstract

Aging is commonly defined as the loss of global homeostasis, which results from progressive alteration of all organs function. This model is currently challenged by recent data showing that interventions that extend lifespan do not always increase the overall fitness of the organism. These data suggest the existence of tissue‐specific factors that regulate the pace of aging in a cell‐autonomous manner. Here, we investigated aging of *Caenorhabditis elegans* striated muscles at the subcellular and the physiological level. Our data show that muscle aging is characterized by a dramatic decrease in the expression of genes encoding proteins required for muscle contraction, followed by a change in mitochondria morphology, and an increase in autophagosome number. Myofilaments, however, remain unaffected during aging. We demonstrated that the conserved transcription factor UNC‐120/SRF regulates muscle aging biomarkers. Interestingly, the role of UNC‐120/SRF in the control of muscle aging can be dissociated from its broader effect on lifespan. In *daf‐2/*insulin/IGF1 receptor mutants, which exhibit a delayed appearance of muscle aging biomarkers and are long‐lived, disruption of *unc‐120* accelerates muscle aging but does not suppress the lifespan phenotype of *daf‐2* mutant. Conversely, *unc‐120* overexpression delays muscle aging but does not increase lifespan. Overall, we demonstrate that UNC‐120/SRF controls the pace of muscle aging in a cell‐autonomous manner downstream of the insulin/IGF1 receptor.

## 1 INTRODUCTION

The survival of multicellular organisms relies on their adaptation to environmental and nutritional variations through complex and permanent interactions between tissues. Thus, increasing the lifespan of an organism is thought to reflect the maintenance of overall tissues integrity and function at advanced age (Hansen & Kennedy, 2016; Laplante & Sabatini, 2012; Rando & Chang, 2012; Russell & Kahn, 2007). Based on this concept, assessing aging in model organisms relies mostly on lifespan measurement. However, recent studies demonstrated that the span of good health is not necessarily proportional to the lifespan among strains with distinct genotypes (Podshivalova, Kerr & Kenyon, 2017) and even among isogenic individuals within a population (Zhang et al., 2016).

These data raise the question of a tissue‐specific impact for the numerous genes identified as modifiers of *Caenorhabditis elegans* lifespan. Indeed, while *C. elegans* is commonly used as an aging model, as illustrated by hundreds of genes identified that influence its lifespan, aging at the tissue scale has been largely overlooked (Herndon et al., 2002; Liu et al., 2013; McGee et al., 2011). To further delineate factors that modulate the pace of physiological tissue aging, we aimed at defining the subcellular changes that take place during aging of muscle tissue. We chose the body‐wall muscle cells (BWM) of *C. elegans* that are functionally equivalent to vertebrate skeletal muscles and are required for locomotion (Gieseler, Qadota & Benian, 2016). Similar to mammals, *C. elegans* display progressive loss of mobility that involves muscle (Herndon et al., 2002) and neurotransmission defects (Liu et al., 2013).

Our data show that the loss of physical performance with age is associated with gradual subcellular changes taking place at different time points in muscle and include a drastic transcriptional downregulation of specific muscle genes, progressive mitochondria fragmentation, and autophagic vesicle accumulation while actin–myosin network remains intact. This sequence of events can be accelerated or delayed upon inhibition or upregulation of the conserved transcription factor UNC‐120/SRF, respectively, and this without necessarily affecting lifespan. Finally, we showed that loss of *daf‐2* delays the pace of muscle aging, at least in part, through the upregulation of *unc‐120* transcripts levels.

## 2 RESULTS

### 2.1 Time course of muscle changes associated with loss of mobility during aging

To identify potential cellular hallmarks of muscle aging, we analyzed worms at different time points during aging, starting from young adults until day 12, when around 50% of worms showed loss of locomotion (Herndon et al., 2002 and Figure S1).

**Figure 1:**
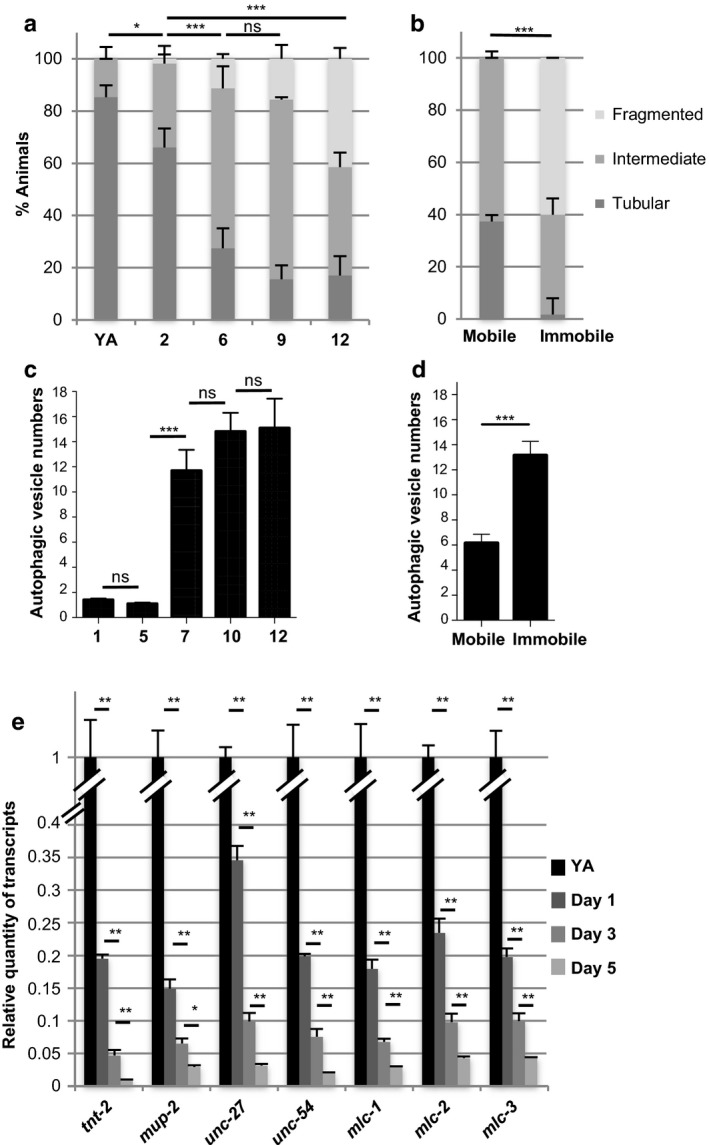
Time course quantification of muscle changes associated with loss of mobility with aging. (a–b) Quantification of animals with the indicated muscle mitochondria phenotypes at different ages from the young adult (YA) stage (*n *=* *41, 56, 62, 45, and 94 for YA, days 2, 6, 9, and 12, respectively) (a) or at day 9, in worms which have been sorted according to their mobility phenotype (*n *=* *59 and 60 for mobile and immobile worms, respectively) (b). Bars are mean ± *SEM*, chi‐square test. (c–d) Quantification of autophagic vesicles number per muscle cell at the indicated ages (*n *=* *35, 40, 48, 63, and 41 for days 1, 5, 7, 10, and 12, respectively) (c) or at day 9 of adulthood, in worms which have been sorted according to their mobility phenotype (*n *=* *38 and 27 for mobile and immobile worms, respectively) (d). Bars are mean ± *SEM*, (c) Kruskal–Wallis and Dunn's post hoc test or (d) Mann–Whitney test. (e) Relative quantity of muscle‐specific gene transcripts at different ages during adulthood. For each gene, levels were normalized to the YA value. Bars are mean ± *SEM* (*n *=* *6), Mann–Whitney test

First, we analyzed mitochondria morphology using transgenic worms that express a mitochondria‐targeted GFP specifically in BWM (Benedetti, Haynes, Yang, Harding & Ron, 2006). As previously published (Regmi, Rolland & Conradt, 2014), we observed the progressive fragmentation of muscle mitochondria with age with a significant decrease in the proportion of animals showing tubular mitochondria as soon as day 2 (Figures 1a and S2a). To analyze whether mitochondria network morphology mirrors the functional status of muscle cells, we separated 9‐day‐old worms according to their locomotion phenotype and scored the mitochondria phenotype in mobile vs. immobile worms. At this age, loss of locomotion was observed in about 20% of worms (Figure S1). Interestingly, the fragmented pattern was clearly restricted to worms showing a loss of locomotion, and tubular mitochondria could only occasionally be observed. By contrast, mobile worms retained either a “young” tubular interconnected or an intermediate pattern (about 40% and 60%, respectively, Figure 1b). These data strongly suggest that mitochondria fragmentation is reflecting loss of muscle function rather than just chronological age.

Second, we analyzed autophagy in aging muscle cells. Previous studies reported the beneficial impact of autophagy on lifespan in *C. elegans* and other species (Madeo, Zimmermann, Maiuri & Kroemer, 2015), and inhibition of autophagy was recently shown to enhance mitochondria dysfunction associated with muscle aging in mice (Carnio et al., 2014). To determine whether the autophagic process was altered during muscle aging, we generated transgenic worms expressing GFP::LGG‐1 under the control of the muscle‐specific *dyc‐1S* promoter (Lecroisey et al., 2008) (Figure S2b). LGG‐1, the *C. elegans* orthologue of LC3, changes from diffuse to punctate pattern when autophagy is induced (Melendez et al., 2003). To validate this marker, we used starvation to induce autophagy (Melendez et al., 2003). The mean number of autophagosomes per muscle cell increased by 56% in starved worms compared to well‐fed control animals (Figure S3a), thus validating this transgene as a tool to monitor autophagy in muscle cells. Furthermore, we checked that the lifespan of transgenic worms was not different as compared to wild type (Figure S3b). Under standard conditions, the average number of autophagic vesicles per muscle cells was stable during the first 5 days of adulthood and subsequently increased by more than sixfold between day 5 and day 7 of adulthood (Figure 1c). When 9‐day‐old worms were separated according to their locomotion phenotype, we observed twice as much vesicles in immobile worms compared to worms that were still mobile (Figure 1d). To ensure that the GFP puncta correspond to autophagic vesicles rather than GFP::LGG‐1 aggregates, we constructed a transgenic line expressing a mutant form of GFP::LGG‐1(G116A) which is no longer targeted to autophagosomes (Manil‐Segalen et al., 2014), specifically in muscle.

Worms carrying this transgene showed a diffuse cytoplasmic GFP staining of muscle cells at all ages, thus excluding the possibility that the GFP::LGG‐1 puncta observed in aged worms would correspond to aggregates triggered by general proteostasis imbalance (Figure S3c). Altogether these data show that the increase in autophagosome number correlates with the loss of mobility but appears at later ages as compared to mitochondria network fragmentation.

We next investigated structural features of muscle cells with age by observing actin and myosin myofilaments, which are essential for muscle contraction. Disorganization of actin thin filaments is a hallmark of muscle degeneration occurring under pathological conditions in *C. elegans* (Gieseler, Grisoni & Segalat, 2000). Interestingly, actin filaments did not show any obvious defects in wild‐type animals up to day 13 (*n *=* *82, Figure S2), while at this age, 50% of the animals are immobile (Figure S1).

We also examined the pattern of the myosin heavy‐chain protein MYO‐3, one of the main components of BWM thick filaments. Several previous studies using the RW1596 strain expressing a *Pmyo‐3*:: *GFP::myo‐3* reporter transgene (Campagnola et al., 2002) described alteration in thick filaments integrity within the first week of adulthood. However, careful analysis of these transgenic animals by the Moerman laboratory revealed myofilaments instability as early as L4 stage, probably as a result of GFP‐tagged myosin overexpression (Meissner et al., 2009). To monitor thick filament integrity in a more physiological context, we generated a GFP::MYO‐3 knock‐in strain (KAG420). Using this line, we did not observe any changes in myosin filament organization as far as day 13 (*n* = 88, Figure S2). This suggests that the loss of mobility is not the consequence of myofilament disruption.

In order to extend our analysis to transcriptional changes, we used the results of a microarray study performed in our laboratory to identify genes whose expression level varies with life expectancy (see Supporting information). Focusing on genes predominantly expressed in BWMs according to Wormbase, we identified 28 evolutionarily conserved genes with expression levels varying from twofold to 20‐fold between the YA stage (before egg production started) and day 7 of adulthood (when worms just stopped to lay eggs). The majority of these genes was downregulated (27 out of 28) and encode proteins required for muscle contraction, including core components of the sarcomere such as the subunits of the troponin complex (six of seven encoded by the *C. elegans* genome: *tnt‐2; mup‐2; unc‐27; tni‐1; tni‐3, and pat‐10*), the tropomyosin (*lev‐11*), muscle myosin regulatory light chains (three of five predicted: *mlc‐1; mlc‐2; mlc‐3*), the two myosin heavy chains (*myo‐3* and *unc‐54*), the paramyosin *unc‐15*, but also nonsarcomeric proteins such as the potassium channel *twk‐18*, the calsequestrin *csq‐1* (Figure S4a). Microarray results were validated for 13 genes randomly picked among those showing between fivefold and 20‐fold variation in transcript levels (Figure S4b). Notably, variations did not seem to result from a general sickness of muscle cells as other muscle‐expressed genes did not show significant variations of their transcript levels (Figure S4b). We then asked whether expression levels started to decrease before day 7 of adulthood. To this aim, we used *rrf‐3(pk1426)* mutants which are sterile at 25°C (thus precluding contamination from the embryonic transcriptome) while harboring wild‐type lifespan (Masse et al., 2008) and analyzed transcript levels for seven genes (among those showing the strongest variations). We observed a significant downregulation as early as day 1 of adulthood for all tested genes (Figure 1e). Thus, the downregulation of sarcomeric gene expression could be an early hallmark of muscle aging and may play a causal role in muscle aging.

### 2.2 Role of myogenic transcription factors in the regulation of muscle gene expression during aging

**Figure 2:**
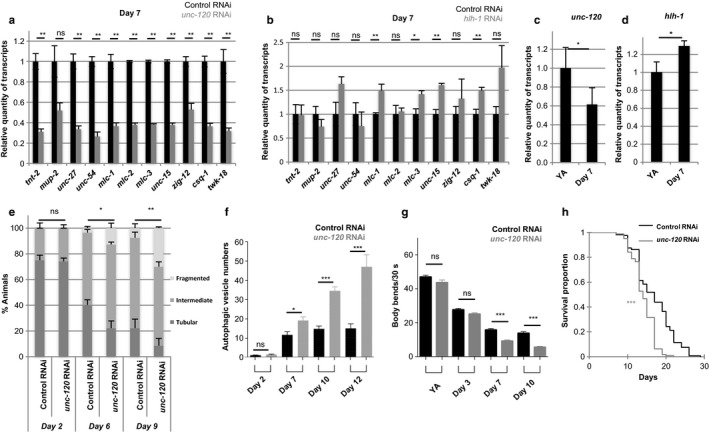
Downregulation of *unc‐120* expression induces precocious appearance of muscle aging markers. (a–b) Relative quantity of transcripts measured in 7‐day‐old animals fed either control or (a) *unc‐120* or (b) *hlh‐1* RNAi bacteria. For each gene, results were normalized to their respective control. Bars are mean ± *SEM* (*n *=* *6), Mann–Whitney test. (c–d) Relative quantity of (c) *unc‐120* or (d) *hlh‐1* transcripts in wild‐type young adults (YA) and 7‐day‐old animals. For each gene, results were normalized to the respective YA stage values. Bars are mean ± *SEM* (*n *=* *6), Mann–Whitney test. (e) Quantification of animals with the indicated muscle mitochondria phenotypes, at days 2, 6, and 9 of adulthood animals expressing a mitochondria‐targeted GFP in muscle cells (see Experimental procedures section), fed control or *unc‐120* RNAi bacteria. *n *=* *52, 57, and 54 for control worms and *n *=* *58, 63, and 70 for *unc‐120* RNAi‐treated worms at days 2, 6, and 9, respectively. Data are presented as mean ± *SEM*, chi‐square test. (f) Number of autophagic vesicles per muscle cell at different days of adulthood in animals expressing a GFP marker of autophagic vesicles in body‐wall muscles (see Experimental procedures section), fed control or *unc‐120* RNAi bacteria. Number of animals scored: *n *=* 40*, 48, 63, and 41 for control worms; *n *=* *44, 50, 50, and 10 for *unc‐120* RNAi‐treated worms at days 2, 7, 10, and 12, respectively. Bars are mean ± *SEM*, Kruskal–Wallis and Dunn's post hoc test. (g) Body bend frequency at different adult ages of animals fed control or *unc‐120* RNAi bacteria. *n *=* *37, 131, 92, and 66 for control worms and *n *=* *34, 126, 94, and 70 for *unc‐120* RNAi‐treated animals at YA, days 3, 7, and 10, respectively. Bars are mean ± *SEM*, Kruskal–Wallis and Dunn's post hoc test. (h) Survival curves of worms fed control or *unc‐120* RNAi bacteria. The corresponding average lifespans were 16.9 ± 0.4 days (*n *=* *137) and 14.1 ± 0.3 days (*n *=* *121) for control and *unc‐120* RNAi conditions, respectively

We hypothesized that myogenic transcription factors may be responsible for the transcript variation observed during early adulthood. The three transcription factors UNC‐120/SRF, HLH‐1/MYOD, and HND‐1/HAND1 are required for BWM differentiation during embryogenesis (Fukushige, Brodigan, Schriefer, Waterston & Krause, 2006; Kuntz, Williams, Sternberg & Wold, 2012; Lei et al., 2010). UNC‐120/SRF and HLH‐1/MYOD remain expressed at adult stage, but their role at this age is unknown (McKay et al., 2003). We thus asked whether their inactivation during adulthood might also affect muscle gene expression with age. To this end, we treated worms with RNAi targeting either *hlh‐1* or *unc‐120* genes starting at pre‐adult/L4 stage. After 24 hr, both *hlh‐1* and *unc‐120* transcripts levels were reduced by 40%, and at 7 days of adulthood, they were reduced by 90% and 50%, respectively (Figure S5a,b). In *unc‐120* RNAi‐treated worms, we consistently observed a twofold to fivefold downregulation of muscle transcript levels at day 7 of adulthood, when compared to age‐matched control (Figure 2a) for which we checked that the downregulation in transcript levels with age was still observed (Figure S6). Conversely, *hlh‐1 RNAi* conditions did not cause significant differences with controls except for four genes that showed a weak trend (0.6 fold) toward increased expression (Figure 2b).

We then asked whether *unc‐120* and *hlh‐1* expression varies with age and we indeed observed a twofold decrease in *unc‐120* transcripts during the first week of adulthood while *hlh‐1* expression levels showed a weak but significant increase (Figure 2c,d). Overall these data suggested that UNC‐120 might play a physiological role in the maintenance of muscle gene expression during adulthood, while HLH‐1 does not seem to have much impact.

### 2.3 Downregulation of *unc‐120* expression induced earlier appearance of muscle aging markers

We next asked whether later markers of muscle aging such as mitochondrial network morphology and autophagosome increase were also affected by *unc‐120* RNAi. At day 6 of adulthood, *unc‐120 RNAi* animals already exhibited altered mitochondria morphology with a decreased fraction of worms with interconnected mitochondria and the appearance of animals with fragmented mitochondria (Figure 2e). Thus, the mitochondrial pattern of 6‐day‐old worms treated with *unc‐120* RNAi was more similar to 9‐day‐old than to age‐matched control worms. At day 9 of adulthood, the proportion of worms with fragmented mitochondria was increased by threefold in *unc‐120* RNAi‐treated worms as compared to age‐matched controls. Quantification of autophagic vesicles in muscle also revealed a stronger increase in vesicles number in *unc‐120* RNAi‐treated worms compared to age‐matched control worms (Figure 2f), while worms expressing the GFP::LGG‐1(G116A) mutant did not show aggregate formation under the same experimental condition (*n* = 19, 37, and 51 at days 3, 7, and 10, respectively, data not shown).

These data strongly suggest that *unc‐120* RNAi causes precocious muscle aging, which might cause the decline of muscle function at younger age during adulthood. To address this hypothesis, we evaluated the capacity of worms to perform body bends in liquid medium. As previously reported (Bansal, Zhu, Yen & Tissenbaum, 2015), control worms showed a progressive age‐related decline in body bend frequency. This decline was enhanced in *unc‐120 RNAi* worms, starting from day 7 of adulthood (Figure 2g). We finally asked whether *unc‐120* downregulation affects longevity and we indeed observed a reduction in average lifespan by 16% (Figure 2h).

Overall our results showed that inhibition of *unc‐120* expression accelerates the appearance of several markers of muscle aging and strongly suggested that *unc‐120* is required for the maintenance of muscle integrity and function during aging.

### 2.4 Muscle‐specific *unc‐120* overexpression is sufficient to delay markers of muscle aging without extending lifespan

**Figure 3:**
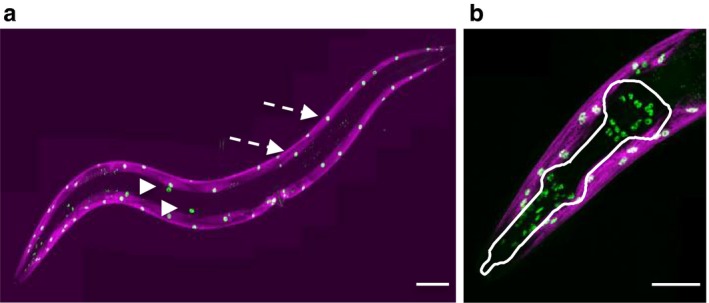
GFP::UNC‐120 expression pattern. (a) Confocal images of a transgenic *gfp::unc‐120* expressing animal. The GFP::UNC‐120 protein is detected in the nuclei of all striated body‐wall muscles cells (two are indicated by dashed arrows) and of the gonadal sheath cells (arrowheads). Body‐wall muscles also express a *troponin::SL2::RFP* knock‐in reporter transgene (red) (see Experimental Procedure). Scale bar: 100 μm. (b) Enlargement of the head region showing the body‐wall muscle nuclei at the periphery and cells nuclei in the pharyngeal region (delineated in white, see text for detailed description). Scale bar: 50 μm

Because *unc‐120* downregulation reduces lifespan *unc‐120* might regulate the pace of muscle aging through systemic mechanisms and/or in a tissue‐specific manner. To further explore UNC‐120 function in muscle aging, we characterized its tissue expression during adulthood using a *GFP::unc‐120* knock‐in line (Supporting information). GFP::UNC‐120 expression was observed in the 95 BWM that run along the anterior–posterior axis, but also in the 20 gonadal sheath cells and in 55 cells in the head (37 pharyngeal muscle cells, nine marginal cells, and nine pharyngeal epithelial cells, Figure 3a,b). In agreement with *unc‐120* transcript variations with age (Figure 2c), we observed a decrease in UNC‐120 protein levels during aging (Figure S7).

**Figure 4:**
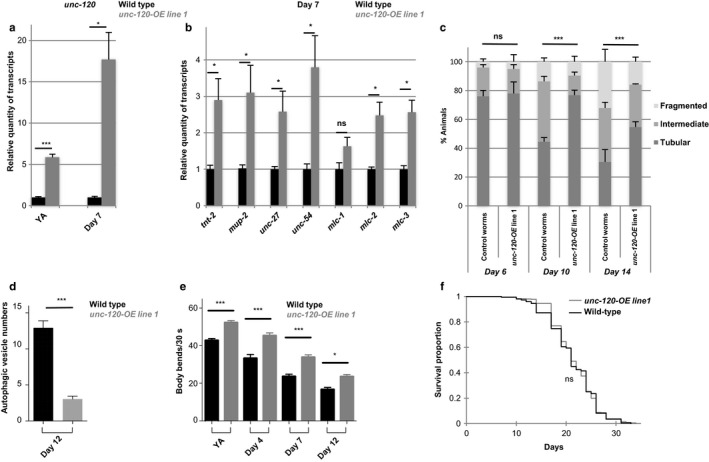
Muscle‐specific *unc‐120* over‐expression is sufficient to delay muscle aging without extending lifespan. (a–b) Relative quantity of (a) *unc‐120* or (b) muscle genes transcripts measured in wild‐type (WT) or *unc‐120* overexpressing worms (*unc‐120‐OE)* at young adult (YA) stage and at day 7 of adulthood. For each gene, results were normalized to the WT level and expressed in relative units. Bars are mean ± *SEM* (*n *=* *8 and 4, respectively, for YA and day 7 of adulthood), Mann–Whitney test. (c) Quantification of animals with the indicated muscle mitochondria phenotypes among WT or *unc‐120* overexpressing worms (*unc‐120‐OE)* at different ages during adulthood. *n *=* *100, 123, and 93 for control worms and *n *=* *100, 117, and 121 for *unc‐120‐OE* worms, at day 6, 10, and 14, respectively. Data are presented as mean ± *SEM*, chi‐square test. (d) Number of autophagic vesicles per muscle cell at day 12 of adulthood in WT or *unc‐120* overexpressing worms (*unc‐120‐OE)*. Number of animals scored: *n = 77* and *79* for WT and *unc‐120‐OE* worms, respectively. Data are mean ± *SEM*, Mann–Whitney test. (e) Body bend frequency of WT or *unc‐120* overexpressing worms (*unc‐120‐OE)* at different ages during adulthood. *n *=* *140, 58, 108, and 112 for control worms and *n *=* *123, 86, 152, and 130 for *unc‐120‐OE* worms, at YA stage and days 4, 7, and 12, respectively. Bars are mean ± *SEM*, Kruskal–Wallis and Dunn's post hoc test. (f) Survival curves of WT or *unc‐120* overexpressing worms (*unc‐120‐OE)*. The corresponding average lifespans were 21.2 ± 0.3 days (*n *=* *245) and 21.6 ± 0.2 days (*n *=* *372) for WT and *unc‐120‐OE* worms, respectively. Five independent experiments have been pooled

We then investigated whether *unc‐120* over‐expression in BWM only was sufficient to modulate muscle aging. *unc‐120* over‐expression (OE) in BWM (see Supporting information, Figures 4a and S8a) induced an increase in the expression of muscle genes in 7‐day‐old adults by at least twofold for most of the genes tested (Figures 4b and S8).

Furthermore, mitochondria morphology remained tubular for longer time and accumulation of autophagic vesicles was prevented in those animals (Figure 4c,d). *unc‐120* OE in muscle was also sufficient for improving worms physical performance until late age as revealed by a 40% increase in the frequency of body bends in 12‐day‐old worms as compared to control (Figures 4e and S8c).

We then asked whether the beneficial effect of *unc‐120* OE on muscle aging markers could result from a broader impact on lifespan (Demontis, Piccirillo, Goldberg & Perrimon, 2013). Remarkably, independent transgenic strains showed either no impact (Figure 4f) or even a 20% reduction in average lifespan (Figure S8d) while they all demonstrated a beneficial impact of *unc‐120* overexpression on muscle aging markers. Overall these results showed that *unc‐120* functions in a cell‐autonomous manner to control muscle aging independently of lifespan regulation.

### 2.5 UNC‐120 is a novel effector of the DAF‐2 insulin/IGF‐1 receptor pathway

The DAF‐2 insulin/IGF‐1 receptor pathway is the most universal regulator of lifespan across species. We thus asked whether muscle aging biomarkers were delayed in long‐lived *daf‐2(e1370)* mutants. We observed a delay in the loss of physical performance after the first week of adulthood in those mutants compared to wild‐type (Figure S9a) in agreement with previously published data (Hahm et al., 2015; Mulcahy, Holden‐Dye & O'Connor, 2013). We next assessed muscle‐specific changes. The expression of sarcomeric genes was increased by two to eightfold at day 5 of adulthood as compared to wild‐type. This increase was observed as early as day 1 of adulthood, while at young adult stage, the mRNA levels of all but one (*tnt‐2*) assessed muscle‐specific genes were not affected in *daf‐2* mutants (Figure S9b–d). Analysis of muscle mitochondria pattern demonstrated a delayed mitochondria fragmentation and only a modest increase in autophagic vesicle number with age in *daf‐2* mutants (Figure S8e–f). Overall, these observations support the hypothesis that *daf‐2* mutations delay the pace of muscle aging.

**Figure 5:**
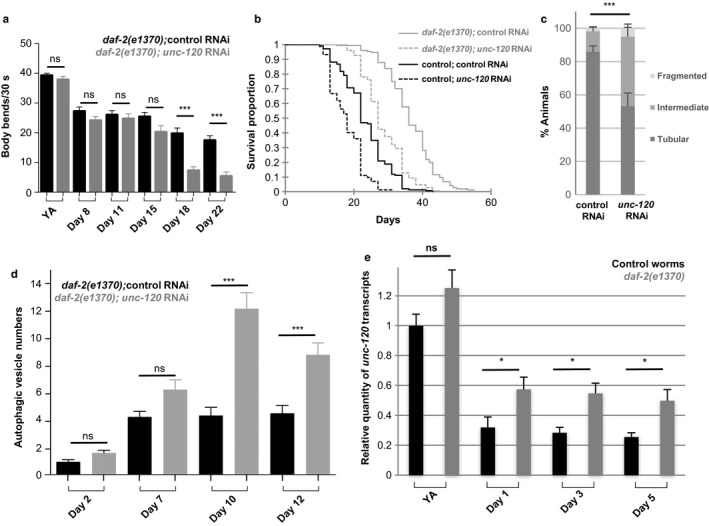
Inhibition of *unc‐120* expression counteracts *daf‐2* beneficial impact on muscle aging. (a) Body bend frequency of *rrf‐3(pk1426); daf‐2(e1370)* mutant worms fed either control or *unc‐120* RNAi bacteria. Data from two independent experiments have been pooled (number of animals scored at young adult [YA] stage and at days 8, 11, 15, 18, and 22 of adulthood: 66, 65, 77, 70, 63, and 72 for control RNAi; 56, 53, 60, 50, 68, and 45 for *unc‐120 RNAi*, respectively) and presented as mean ± *SEM*, Kruskal–Wallis and Dunn's post hoc test. (b) Survival curves of control (black) and *daf‐2(e1370)* mutant (grey) worms fed control or *unc‐120* RNAi bacteria. Two independent experiments have been pooled. The corresponding average lifespans were 23.5 ± 0.6 days (*n *=* *143) and 17.9 ± 0.4 days (*n *=* *145) for control worms on control or *unc‐120 RNAi* bacteria, respectively, and 36.5 ± 0.6 days (*n *=* *146) and 28.5 ± 0.5 days (*n *=* *149) for *daf‐2(e1370)* worms on control or *unc‐120* RNAi bacteria, respectively. (c) Quantification of animals with the indicated muscle mitochondria phenotypes at day 9 of adulthood among *daf‐2(e1370)* worms fed control or *unc‐120* RNAi bacteria. Number of animals scored: *n *=* *74 and 91 for worms on control and *unc‐120* RNAi*,* respectively. Data are presented as mean ± *SEM*, chi‐square test. (d) Number of autophagic vesicles per muscle cell at different days of adulthood in *daf‐2(e1370)* worms expressing a GFP marker of autophagic vesicles in muscle, fed control, or *unc‐120* RNAi bacteria. Number of animals scored: *n = 42*, 48, 63, and 41 for control worms; *n = *48, 54, 60, and 50 for *unc‐120* RNAi‐treated worms at days 2, 7, 10, and 12, respectively. Data are presented as mean ± *SEM*, Kruskal–Wallis and Dunn's post hoc test. (e) Relative quantity of *unc‐120* transcripts measured in *rrf‐3(pk1426)* control or *rrf‐3(pk1426); daf‐2(e1370)* worms at YA stage and at different days of adulthood. For each gene, results were normalized to the respective control. Bars are mean ± *SEM* (*n *=* *6), Mann–Whitney test

We then asked whether the beneficial effect of *daf‐2* mutation may be dependent on *unc‐120*. Inhibition of *unc‐120* expression in *daf‐2* mutants caused a dramatic decreased of physical performance at day 18 (Figure 5a). This reduction can unlikely be explained by a systemic effect of *unc‐120* inactivation because at that stage, 100% of the *daf‐2(e1370); unc‐120* RNAi worms are alive (Figure 5b). We thus addressed whether *unc‐120* downregulation also impacts the mitochondria and autophagy phenotypes with age. We indeed observed a significant decrease in the percentage of worms with interconnected mitochondria (Figure 5c) and a significant increase in autophagic vesicles number (Figure 5d). These data thus strongly suggest that *unc‐120* is required, at least in part, in *daf‐2* mutants to delay the loss of mobility while *daf‐2* and *unc‐120* may act independently to regulate lifespan. As *unc‐120* expression is downregulated with physiological aging, we asked whether *daf‐2* mutation may prevent its downregulation. We indeed observed a twofold increase in *unc‐120* transcripts levels in *daf‐2* mutants as early as day 1 of adulthood (Figure 5e) suggesting that the genetic interaction between *daf‐2* and *unc‐120* in the control of muscle aging relies at least in part on *unc‐120* transcriptional regulation.

## 3 DISCUSSION

A number of factors have been proposed to be involved in muscle aging in mammals (including mitochondria defects and imbalanced proteostasis (Marzetti et al., 2013; Carnio et al., 2014)). However, studies analyzed experimentally induced muscle atrophy models, which may imply different mechanisms responsible for muscle dysfunction as compared to physiological aging. Our study establishes a sequence of subcellular and molecular events that occur during muscle aging and that are associated with the loss of mobility with age.

### 3.1 Mitochondria alteration precedes autophagy deregulation in aging muscle

Age‐dependent muscle degeneration involves a stereotyped sequence of events in which mitochondria modification constitutes the first morphological modification observed. Mitochondria are dynamic organelles whose morphology depends on different factors including their movement along cytoskeletal tracks, their fusion and fission (Otera & Mihara, 2011). Increased fragmentation of mitochondrial network is usually associated with impaired mitochondria that will be targeted for degradation by autophagy (Twig & Shirihai, 2011). Our data showed that mitochondria fragmentation appeared between day 2 and day 6 of adulthood when the decrease in physical performance was first observed (Figure 2g). Similar changes in mitochondria morphology have been reported by Regmi et al. (2014) using a different reporter strain, while they also showed that the mitochondria pattern at day 5 or day 7 of adulthood was not predictive of animal lifespan. Interestingly, our data strongly suggest that it is rather predictive of muscle health span, as mitochondria fragmentation correlated with loss of mobility (Figure 1b), and that it can be considered as an early marker of muscle fitness.

Our data show that autophagy is deregulated in muscle with age and that the increase in autophagosomes is more important in immobile vs. mobile worms of the same age. This increase could be due to an enhancement or a blockade of autophagy. Recent results (Chang, Kumsta, Hellman, Adams & Hansen, 2017) described a similar kinetics in the increase in autophagosomes number with age, using a line in which mCherry::GFP::LGG‐1 was expressed under the control of its own promoter. Interestingly, in the presence of bafilomycin A (an inhibitor of lysosomal acidification which blocks autophagy flux), the pool size of autophagic vesicles did not increase much in day 7 and older animals as compared to younger adults, thus strongly suggesting that a blockade of the autophagy flux takes place with aging (Chang et al., 2017).

Yet the increase in autophagosomes number is taking place several days after the onset of physical performance decline. In agreement with those data, a previous study reported that in muscle tissue, the rupture of protein homeostasis was not detectable before day 12 of adulthood, as revealed by aggregates formation of a conformation unstable firefly luciferase mutant (Gupta et al., 2011). Overall those observations suggest that alteration of autophagy does not appear as a primary cause but rather as a consequence of muscle aging.

### 3.2 Early downregulation of muscle gene expression

The earliest muscle‐specific change that we observed is the dramatic downregulation in the expression of genes encoding proteins required for muscle contraction, as soon as day 1 of adulthood. These results provide mechanistic insights that substantiate the proteomic data previously published by Liang et al. (2014) who used an unbiased global proteomics approach to investigate proteomic variations in worms with age. They reported that between day 1 and day 5 of adulthood the amount of numerous sarcomeric proteins decreases, including myosin heavy‐chain isoforms (MYO‐1, MYO‐2, MYO‐3, UNC‐54), myosin light‐chain isoforms (MLC‐1, MLC‐3), troponin T (MUP‐2), tropomyosin (LEV‐11), and paramyosin (UNC‐15), mitochondrial creatine kinase (W10C8.5) and calsequestrin (CSQ‐1), which we all found to be transcriptionally downregulated. Interestingly, a second proteomic study reported an increased quantity of several of those proteins in *daf‐2* mutants (Depuydt et al., 2013). In light of our results, those changes in protein levels may result at least in part from the impact of *daf‐2* on transcripts levels of the corresponding genes (Figure S8b–d). Furthermore, proteins turnover with age was also recently investigated by Dhondt et al. (2016) and revealed that, while some proteins like the myosin heavy‐chain UNC‐54 exhibited an extremely long half‐live (298 hr), others, such as the paramyosin UNC‐15, showed much higher turnover (66 hr). Therefore, the substantial transcriptional changes that we reported during early adulthood will most probably have different consequence on muscle integrity depending on the stability and function of the corresponding protein.

How this sequence of events is related to pathologic muscle degeneration? Progressive muscle degeneration is also observed in the context of genetic disorders such as muscular dystrophy. Interestingly, *daf‐2* mutation was shown to counteract muscle defects associated with dystrophin mutation (Oh & Kim, 2013). Furthermore, mitochondria fragmentation has also been involved in muscle degeneration in a model of Duchenne muscular dystrophy in *C. elegans,* which combines mutations of both *hlh‐1/myoD* and *dys‐1/dystrophin* (Giacomotto et al., 2013; Gieseler et al., 2000). However, in this genetic context, mitochondria fragmentation occurs well before any obvious sign of degeneration can be detected (Giacomotto et al., 2013). Additionally, dystrophic mutants are also characterized by a strong disorganization of actin cytoskeleton, early accumulation of autophagosomes, and the loss of muscle cells (Brouilly et al., 2015). In contrast during physiological aging of wild‐type worms, actin and myosin filaments remain essentially intact in worms with complete loss of mobility and accumulation of autophagosomes is a late event. Thus, although similar subcellular features are observed in pathologic and age‐dependent muscle degeneration, the sequence of events that take place during muscle aging is different. Those observations strongly suggest that dystrophic models and muscle aging involve distinct mechanisms.

### 3.3 UNC‐120/SRF is required for muscle maintenance during adulthood

Our data uncover a novel role for *unc‐120* in muscle maintenance during adulthood. Interestingly, while myogenesis requires *hlh‐1* and involves the direct positive transcriptional regulation of *unc‐120* by HLH‐1, HLH‐1 does not seem to play an essential role in the regulation of the different muscle aging biomarkers (Figure 2b and data not shown). Conversely, *unc‐120* transcript levels decrease with age and precocious downregulation of *unc‐120* expression further decreases sarcomeric genes expression, accelerates mitochondria fragmentation, autophagic vesicle accumulation, and is associated with premature loss of mobility. Furthermore, *unc‐120* overexpression is sufficient to delay muscle aging.

The function of UNC‐120 in muscle aging may involve the regulation of the expression of genes required for mitochondria dynamics and autophagy activity. Likewise in *Drosophila*, FOXO muscle‐specific overexpression is sufficient to delay muscle functional decay, and this effect relies at least in part on the increased expression of several core components of autophagosomes which appears to be sufficient for maintaining basal autophagy with age (Demontis & Perrimon, 2010). Further investigation of UNC‐120 transcriptional targets should help to decipher its mechanism of action.

UNC‐120 is homologous to the mammalian MADS box family transcription factor SRF (for serum response factor). Similar to UNC‐120, SRF is essential for muscle development and muscle‐specific gene expression in mice (Fukushige et al., 2006; Li et al., 2005). Selective SRF knockout in adult postmitotic myofibers is responsible for premature muscle atrophy associated with different features that are reminiscent of defects observed in aging skeletal muscle. Age‐associated decrease in SRF expression was also observed in both mice and human muscles (Lahoute et al., 2008). Our data suggest that the age‐associated physiological decrease in UNC‐120/SRF expression contributes to different muscle changes associated with muscle aging and that this mechanism may be conserved through species.

### 3.4 Role of the DAF‐2/insulin‐IGF‐1 pathway in muscle aging

Our data show that *daf‐2* mutants exhibit delayed appearance of different biomarkers of muscle aging and support the hypothesis that the health span of this tissue is extended in these mutants. The dramatic increase in autophagy vesicles observed during aging is strongly suppressed in *daf‐2* mutants. Chang et al. (2017) also reported an absence of increase in autophagy vesicles in old *daf‐2* mutants, although the number of vesicles was high during early adulthood. This apparent discrepancy might be related to the different promoters used to express LGG‐1 reporters in the two studies. Our results also strongly suggest that *daf‐2* impacts muscle aging at least in part through the control of *unc‐120* transcript levels. Indeed, *daf‐2* mutants show higher levels of *unc‐120* transcripts, and inhibiting *unc‐120* expression by RNAi in *daf‐2* mutants restores wild‐type levels of autophagic vesicles numbers, mitochondria fragmentation, and mobility.

However, under the same experimental conditions, downregulating *unc‐120* expression in *daf‐2* mutants is not sufficient to recapitulate wild‐type lifespan. Thus, our data strongly suggest that the control of muscle fitness by DAF‐2 through UNC‐120 can be dissociated from its function in overall lifespan. In agreement with this hypothesis, *unc‐120* overexpression increases mobility at late age without extending lifespan.

Several reports described the cell nonautonomous impact of different genes on lifespan and support a model where aging rates between tissues are interdependent (Chang et al., 2017; Durieux, Wolff & Dillin, 2011; Laplante & Sabatini, 2012; Rando & Chang, 2012; Russell & Kahn, 2007).

Furthermore, genes that delay motility decline also affect lifespan in both *C. elegans*, such as *hpa‐1* and *hpa‐2* (Iwasa, Yu, Xue & Driscoll, 2010), and in *Drosophila* as exemplified by FOXO muscle‐specific overexpression (Demontis & Perrimon, 2010).

Our results, however, strongly suggest that the pace of aging of a specific organ does not necessarily impact overall lifespan. Large‐scale screening for genetic modifiers of aging was essentially based on the *C. elegans* lifespan phenotype. Our results, which dissociate the regulation of muscle aging from lifespan, show that future screen focused on tissue‐specific features should uncover novel aging regulators.

## 4 EXPERIMENTAL PROCEDURES

### 4.1 *C. elegans* strains and media

All strains were maintained at 20°C (unless otherwise indicated) on nematode growth medium agar plates freshly poured and seeded with *Escherichia coli* strain OP50 culture. The wild‐type reference strain was *C. elegans* N2 Bristol. Previously generated lines used in this study were as follows: NL2099 *rrf‐3(pk1426)* (Simmer et al., 2002), CB1370 *daf‐2(e1370)* (Kenyon, Chang, Gensch, Rudner & Tabtiang, 1993), and SJ4103 *zcIs14* [Pmyo‐3::GFP(mitochondria)] (Benedetti et al., 2006). The transgenic lines created for this work are described in supporting information.

Processing of transgenic strains for mitochondria, autophagic vesicles, and myofilament analysis was performed as described in supporting information.

### 4.2 RNAi feeding and lifespan assays

Bacterial feeding RNAi experiments and lifespan assay were carried out as described previously (Masse et al., 2008). Further details are in supporting information.

### 4.3 Body bend frequency assays

Body bend frequency assay was performed as described in supporting information.

### 4.4 Reverse transcription–qPCR

Transcript‐level analysis was performed as described in supporting information.

### 4.5 Identification of muscle‐specific genes whose expression varies with lifespan expectancy

Muscle genes whose expression varies with lifespan expectancy were identified from a microarray approach described in supporting information.

### 4.6 Statistical analysis

Statistical analyses were performed as described in supporting information.

## CONFLICT OF INTEREST

None declared.

## AUTHOR CONTRIBUTIONS

A.MdL., L.M., and F.S. conceived, designed the study, performed the experimental works, and wrote the manuscript. A.MdL., L.M., F.S., and J.L.B analyzed the data. L. P. and M.C.M. generated and validated the muscle‐specific autophagy reporter strains. A.MdL., L.M., K.G., J.L.B., and F.S. edited the manuscript.

## References

[acel12713-bib-0001] Bansal, A. , Zhu, L. J. , Yen, K. , & Tissenbaum, H. A. (2015). Uncoupling lifespan and healthspan in *Caenorhabditis elegans* longevity mutants. Proceedings of the National Academy of Sciences of the United States of America, 112, E277–E286. https://doi.org/10.1073/pnas.1412192112 25561524 10.1073/pnas.1412192112 PMC4311797

[acel12713-bib-0002] Benedetti, C. , Haynes, C. M. , Yang, Y. , Harding, H. P. , & Ron, D. (2006). Ubiquitin‐like protein 5 positively regulates chaperone gene expression in the mitochondrial unfolded protein response. Genetics, 174, 229–239. https://doi.org/10.1534/genetics.106.061580 16816413 10.1534/genetics.106.061580 PMC1569816

[acel12713-bib-0003] Brouilly, N. , Lecroisey, C. , Martin, E. , Pierson, L. , Mariol, M. C. , Qadota, H. , … Gieseler, K. (2015). Ultra‐structural time‐course study in the *C. elegans* model for Duchenne muscular dystrophy highlights a crucial role for sarcomere‐anchoring structures and sarcolemma integrity in the earliest steps of the muscle degeneration process. Human Molecular Genetics, 24, 6428–6445. https://doi.org/10.1093/hmg/ddv353 26358775 10.1093/hmg/ddv353

[acel12713-bib-0004] Campagnola, P. J. , Millard, A. C. , Terasaki, M. , Hoppe, P. E. , Malone, C. J. , & Mohler, W. A. (2002). Three‐dimensional high‐resolution second‐harmonic generation imaging of endogenous structural proteins in biological tissues. Biophysical Journal, 82, 493–508. https://doi.org/10.1016/S0006-3495(02)75414-3 11751336 10.1016/S0006-3495(02)75414-3 PMC1302489

[acel12713-bib-0005] Carnio, S. , LoVerso, F. , Baraibar, M. A. , Longa, E. , Khan, M. M. , Maffei, M. , … Sandri, M. (2014). Autophagy impairment in muscle induces neuromuscular junction degeneration and precocious aging. Cell Reports, 8, 1509–1521. https://doi.org/10.1016/j.celrep.2014.07.061 25176656 10.1016/j.celrep.2014.07.061 PMC4534571

[acel12713-bib-0006] Chang, J. T. , Kumsta, C. , Hellman, A. B. , Adams, L. M. , Hansen, M. (2017). Spatiotemporal regulation of autophagy during *Caenorhabditis elegans* aging. Elife 6, pii: e18459. 10.7554/eLife.18459 PMC5496740 28675140

[acel12713-bib-0007] Demontis, F. , & Perrimon, N. (2010). FOXO/4E‐BP signaling in *Drosophila* muscles regulates organism‐wide proteostasis during aging. Cell, 143, 813–825. https://doi.org/10.1016/j.cell.2010.10.007 21111239 10.1016/j.cell.2010.10.007 PMC3066043

[acel12713-bib-0008] Demontis, F. , Piccirillo, R. , Goldberg, A. L. , & Perrimon, N. (2013). Mechanisms of skeletal muscle aging: Insights from *Drosophila* and mammalian models. Disease Models & Mechanisms, 6, 1339–1352. https://doi.org/10.1242/dmm.012559 24092876 10.1242/dmm.012559 PMC3820258

[acel12713-bib-0009] Depuydt, G. , Xie, F. , Petyuk, V. A. , Shanmugam, N. , Smolders, A. , Dhondt, I. , … Braeckman, B. P. (2013). Reduced insulin/insulin‐like growth factor‐1 signaling and dietary restriction inhibit translation but preserve muscle mass in *Caenorhabditis elegans* . Molecular & Cellular Proteomics: MCP, 12, 3624–3639. https://doi.org/10.1074/mcp.M113.027383 24002365 10.1074/mcp.M113.027383 PMC3861712

[acel12713-bib-0010] Dhondt, I. , Petyuk, V. A. , Cai, H. , Vandemeulebroucke, L. , Vierstraete, A. , Smith, R. D. , … Braeckman, B. P. (2016). FOXO/DAF‐16 activation slows down turnover of the majority of proteins in *C. elegans* . Cell Reports, 16, 3028–3040. https://doi.org/10.1016/j.celrep.2016.07.088 27626670 10.1016/j.celrep.2016.07.088 PMC5434875

[acel12713-bib-0011] Durieux, J. , Wolff, S. , & Dillin, A. (2011). The cell‐non‐autonomous nature of electron transport chain‐mediated longevity. Cell, 144, 79–91. https://doi.org/10.1016/j.cell.2010.12.016 21215371 10.1016/j.cell.2010.12.016 PMC3062502

[acel12713-bib-0012] Fukushige, T. , Brodigan, T. M. , Schriefer, L. A. , Waterston, R. H. , & Krause, M. (2006). Defining the transcriptional redundancy of early bodywall muscle development in *C. elegans*: Evidence for a unified theory of animal muscle development. Genes & Development, 20, 3395–3406. https://doi.org/10.1101/gad.1481706 17142668 10.1101/gad.1481706 PMC1698447

[acel12713-bib-0013] Giacomotto, J. , Brouilly, N. , Walter, L. , Mariol, M. C. , Berger, J. , Segalat, L. , … Gieseler, K. (2013). Chemical genetics unveils a key role of mitochondrial dynamics, cytochrome c release and IP3R activity in muscular dystrophy. Human Molecular Genetics, 22, 4562–4578. https://doi.org/10.1093/hmg/ddt302 23804750 10.1093/hmg/ddt302

[acel12713-bib-0014] Gieseler, K. , Grisoni, K. , & Segalat, L. (2000). Genetic suppression of phenotypes arising from mutations in dystrophin‐related genes in *Caenorhabditis elegans* . Current Biology, 10, 1092–1097. https://doi.org/10.1016/S0960-9822(00)00691-6 10996789 10.1016/s0960-9822(00)00691-6

[acel12713-bib-0015] Gieseler, K. , Qadota, H. , Benian, G. M. (2016). Development, structure, and maintenance of *C. elegans* body wall muscle. WormBook, 2016, 1–59. https://doi.org/10.1895/wormbook.1.81.2 10.1895/wormbook.1.81.2 PMC5410635 27555356

[acel12713-bib-0016] Gupta, R. , Kasturi, P. , Bracher, A. , Loew, C. , Zheng, M. , Villella, A. , … Raychaudhuri, S. (2011). Firefly luciferase mutants as sensors of proteome stress. Nature Methods, 8, 879–884. https://doi.org/10.1038/nmeth.1697 21892152 10.1038/nmeth.1697

[acel12713-bib-0017] Hahm, J. H. , Kim, S. , DiLoreto, R. , Shi, C. , Lee, S. J. , Murphy, C. T. , & Nam, H. G. (2015). *C. elegans* maximum velocity correlates with healthspan and is maintained in worms with an insulin receptor mutation. Nature Communications, 6, 8919 https://doi.org/10.1038/ncomms9919 10.1038/ncomms9919 PMC4656132 26586186

[acel12713-bib-0018] Hansen, M. , & Kennedy, B. K. (2016). Does longer lifespan mean longer healthspan? Trends in Cell Biology, 26, 565–568. https://doi.org/10.1016/j.tcb.2016.05.002 27238421 10.1016/j.tcb.2016.05.002 PMC4969078

[acel12713-bib-0019] Herndon, L. A. , Schmeissner, P. J. , Dudaronek, J. M. , Brown, P. A. , Listner, K. M. , Sakano, Y. , … Driscoll, M. (2002). Stochastic and genetic factors influence tissue‐specific decline in ageing *C. elegans* . Nature, 419, 808–814. https://doi.org/10.1038/nature01135 12397350 10.1038/nature01135

[acel12713-bib-0020] Iwasa, H. , Yu, S. , Xue, J. , & Driscoll, M. (2010). Novel EGF pathway regulators modulate *C. elegans* healthspan and lifespan via EGF receptor, PLC‐gamma, and IP3R activation. Aging Cell, 9, 490–505. https://doi.org/10.1111/j.1474-9726.2010.00575.x 20497132 10.1111/j.1474-9726.2010.00575.x PMC5859306

[acel12713-bib-0021] Kenyon, C. , Chang, J. , Gensch, E. , Rudner, A. , & Tabtiang, R. (1993). A *C. elegans* mutant that lives twice as long as wild type. Nature, 366, 461–464. https://doi.org/10.1038/366461a0 8247153 10.1038/366461a0

[acel12713-bib-0022] Kuntz, S. G. , Williams, B. A. , Sternberg, P. W. , & Wold, B. J. (2012). Transcription factor redundancy and tissue‐specific regulation: Evidence from functional and physical network connectivity. Genome Research, 22, 1907–1919. https://doi.org/10.1101/gr.133306.111 22730465 10.1101/gr.133306.111 PMC3460186

[acel12713-bib-0023] Lahoute, C. , Sotiropoulos, A. , Favier, M. , Guillet‐Deniau, I. , Charvet, C. , Ferry, A. , … Daegelen, D. (2008). Premature aging in skeletal muscle lacking serum response factor. PLoS ONE, 3, e3910 https://doi.org/10.1371/journal.pone.0003910 19079548 10.1371/journal.pone.0003910 PMC2593784

[acel12713-bib-0024] Laplante, M. , & Sabatini, D. M. (2012). mTOR signaling in growth control and disease. Cell, 149, 274–293. https://doi.org/10.1016/j.cell.2012.03.017 22500797 10.1016/j.cell.2012.03.017 PMC3331679

[acel12713-bib-0025] Lecroisey, C. , Martin, E. , Mariol, M. C. , Granger, L. , Schwab, Y. , Labouesse, M. , … Gieseler, K. (2008). DYC‐1, a protein functionally linked to dystrophin in *Caenorhabditis elegans* is associated with the dense body, where it interacts with the muscle LIM domain protein ZYX‐1. Molecular Biology of the Cell, 19, 785–796. 18094057 10.1091/mbc.E07-05-0497 PMC2262962

[acel12713-bib-0026] Lei, H. , Fukushige, T. , Niu, W. , Sarov, M. , Reinke, V. , & Krause, M. (2010). A widespread distribution of genomic CeMyoD binding sites revealed and cross validated by ChIP‐Chip and ChIP‐Seq techniques. PLoS ONE, 5, e15898 https://doi.org/10.1371/journal.pone.0015898 21209968 10.1371/journal.pone.0015898 PMC3012110

[acel12713-bib-0027] Li, S. , Czubryt, M. P. , McAnally, J. , Bassel‐Duby, R. , Richardson, J. A. , Wiebel, F. F. , … Olson, E. N. (2005). Requirement for serum response factor for skeletal muscle growth and maturation revealed by tissue‐specific gene deletion in mice. Proceedings of the National Academy of Sciences of the United States of America, 102, 1082–1087. https://doi.org/10.1073/pnas.0409103102 15647354 10.1073/pnas.0409103102 PMC545866

[acel12713-bib-0028] Liang, V. , Ullrich, M. , Lam, H. , Chew, Y. L. , Banister, S. , Song, X. , … Nicholas, H. R. (2014). Altered proteostasis in aging and heat shock response in *C. elegans* revealed by analysis of the global and de novo synthesized proteome. Cellular and Molecular Life Sciences, 71, 3339–3361. https://doi.org/10.1007/s00018-014-1558-7 24458371 10.1007/s00018-014-1558-7 PMC4131143

[acel12713-bib-0029] Liu, J. , Zhang, B. , Lei, H. , Feng, Z. , Liu, J. , Hsu, A. L. , & Xu, X. Z. (2013). Functional aging in the nervous system contributes to age‐dependent motor activity decline in *C. elegans* . Cell Metabolism, 18, 392–402. https://doi.org/10.1016/j.cmet.2013.08.007 24011074 10.1016/j.cmet.2013.08.007 PMC3811915

[acel12713-bib-0030] Madeo, F. , Zimmermann, A. , Maiuri, M. C. , & Kroemer, G. (2015). Essential role for autophagy in life span extension. The Journal of Clinical Investigation, 125, 85–93. https://doi.org/10.1172/JCI73946 25654554 10.1172/JCI73946 PMC4382258

[acel12713-bib-0031] Manil‐Segalen, M. , Lefebvre, C. , Jenzer, C. , Trichet, M. , Boulogne, C. , Satiat‐Jeunemaitre, B. , & Legouis, R. (2014). The *C. elegans* LC3 acts downstream of GABARAP to degrade autophagosomes by interacting with the HOPS subunit VPS39. Developmental Cell, 28, 43–55. https://doi.org/10.1016/j.devcel.2013.11.022 24374177 10.1016/j.devcel.2013.11.022

[acel12713-bib-0033] Marzetti, E. , Csiszar, A. , Dutta, D. , Balagopal, G. , Calvani, R. , & Leeuwenburgh, C. (2013). Role of mitochondrial dysfunction and altered autophagy in cardiovascular aging and disease: From mechanisms to therapeutics. American Journal of Physiology. Heart and Circulatory Physiology, 305, H459–H476. https://doi.org/10.1152/ajpheart.00936.2012 23748424 10.1152/ajpheart.00936.2012 PMC3891249

[acel12713-bib-0034] Masse, I. , Molin, L. , Mouchiroud, L. , Vanhems, P. , Palladino, F. , Billaud, M. , & Solari, F. (2008). A novel role for the SMG‐1 kinase in lifespan and oxidative stress resistance in *Caenorhabditis elegans* . PLoS ONE, 3, e3354 https://doi.org/10.1371/journal.pone.0003354 18836529 10.1371/journal.pone.0003354 PMC2556085

[acel12713-bib-0035] McGee, M. D. , Weber, D. , Day, N. , Vitelli, C. , Crippen, D. , Herndon, L. A. , … Melov, S. (2011). Loss of intestinal nuclei and intestinal integrity in aging *C. elegans* . Aging Cell, 10, 699–710. https://doi.org/10.1111/j.1474-9726.2011.00713.x 21501374 10.1111/j.1474-9726.2011.00713.x PMC3135675

[acel12713-bib-0036] McKay, S. J. , Johnsen, R. , Khattra, J. , Asano, J. , Baillie, D. L. , Chan, S. , … Moerman, D. G. (2003). Gene expression profiling of cells, tissues, and developmental stages of the nematode *C. elegans* . Cold Spring Harbor Symposia on Quantitative Biology, 68, 159–169. https://doi.org/10.1101/sqb.2003.68.159 15338614 10.1101/sqb.2003.68.159

[acel12713-bib-0037] Meissner, B. , Warner, A. , Wong, K. , Dube, N. , Lorch, A. , McKay, S. J. , … Moerman, D. G. (2009). An integrated strategy to study muscle development and myofilament structure in *Caenorhabditis elegans* . PLoS Genetics, 5, e1000537. 19557190 10.1371/journal.pgen.1000537 PMC2694363

[acel12713-bib-0038] Melendez, A. , Talloczy, Z. , Seaman, M. , Eskelinen, E. L. , Hall, D. H. , & Levine, B. (2003). Autophagy genes are essential for dauer development and life‐span extension in *C. elegans* . Science, 301, 1387–1391. https://doi.org/10.1126/science.1087782 12958363 10.1126/science.1087782

[acel12713-bib-0040] Mulcahy, B. , Holden‐Dye, L. , & O'Connor, V. (2013). Pharmacological assays reveal age‐related changes in synaptic transmission at the *Caenorhabditis elegans* neuromuscular junction that are modified by reduced insulin signalling. Journal of Experimental Biology, 216, 492–501. https://doi.org/10.1242/jeb.068734 23038730 10.1242/jeb.068734

[acel12713-bib-0041] Oh, K. H. , & Kim, H. (2013). Reduced IGF signaling prevents muscle cell death in a *Caenorhabditis elegans* model of muscular dystrophy. Proceedings of the National Academy of Sciences of the United States of America, 110, 19024–19029. https://doi.org/10.1073/pnas.1308866110 24191049 10.1073/pnas.1308866110 PMC3839758

[acel12713-bib-0042] Otera, H. , & Mihara, K. (2011). Molecular mechanisms and physiologic functions of mitochondrial dynamics. Journal of Biochemistry, 149, 241–251. https://doi.org/10.1093/jb/mvr002 21233142 10.1093/jb/mvr002

[acel12713-bib-0043] Podshivalova, K. , Kerr, R. A. , & Kenyon, C. (2017). How a mutation that slows aging can also disproportionately extend end‐of‐life decrepitude. Cell Reports, 19, 441–450. https://doi.org/10.1016/j.celrep.2017.03.062 28423308 10.1016/j.celrep.2017.03.062 PMC5526670

[acel12713-bib-0044] Rando, T. A. , & Chang, H. Y. (2012). Aging, rejuvenation, and epigenetic reprogramming: Resetting the aging clock. Cell, 148, 46–57. https://doi.org/10.1016/j.cell.2012.01.003 22265401 10.1016/j.cell.2012.01.003 PMC3336960

[acel12713-bib-0045] Regmi, S. G. , Rolland, S. G. , & Conradt, B. (2014). Age‐dependent changes in mitochondrial morphology and volume are not predictors of lifespan. Aging, 6, 118–130. https://doi.org/10.18632/aging.100639 24642473 10.18632/aging.100639 PMC3969280

[acel12713-bib-0046] Russell, S. J. , & Kahn, C. R. (2007). Endocrine regulation of ageing. Nature Reviews Molecular Cell Biology, 8, 681–691. https://doi.org/10.1038/nrm2234 17684529 10.1038/nrm2234

[acel12713-bib-0047] Simmer, F. , Tijsterman, M. , Parrish, S. , Koushika, S. P. , Nonet, M. L. , Fire, A. , … Plasterk, R. H. (2002). Loss of the putative RNA‐directed RNA polymerase RRF‐3 makes *C. elegans* hypersensitive to RNAi. Current Biology, 12, 1317–1319. https://doi.org/10.1016/S0960-9822(02)01041-2 12176360 10.1016/s0960-9822(02)01041-2

[acel12713-bib-0048] Twig, G. , & Shirihai, O. S. (2011). The interplay between mitochondrial dynamics and mitophagy. Antioxidants & Redox Signaling, 14, 1939–1951. https://doi.org/10.1089/ars.2010.3779 21128700 10.1089/ars.2010.3779 PMC3078508

[acel12713-bib-0050] Zhang, W. B. , Sinha, D. B. , Pittman, W. E. , Hvatum, E. , Stroustrup, N. , & Pincus, Z. (2016). Extended Twilight among isogenic *C. elegans* causes a disproportionate scaling between lifespan and health. Cell Systems, 3, 333–345.e334. https://doi.org/10.1016/j.cels.2016.09.003 27720632 10.1016/j.cels.2016.09.003 PMC5111811

