# Supplementary material for: UNC‐120/SRF independently controls muscle aging and lifespan in Caenorhabditis elegans
Source: Aging Cell. 2018 Jan 3;17(2):e12713. doi: 10.1111/acel.12713 (PMC5847867; doi:10.1111/acel.12713)
Supplement: Supplementary file 1 [file ACEL-17-e12713-s001.pptx]

## Slide 1
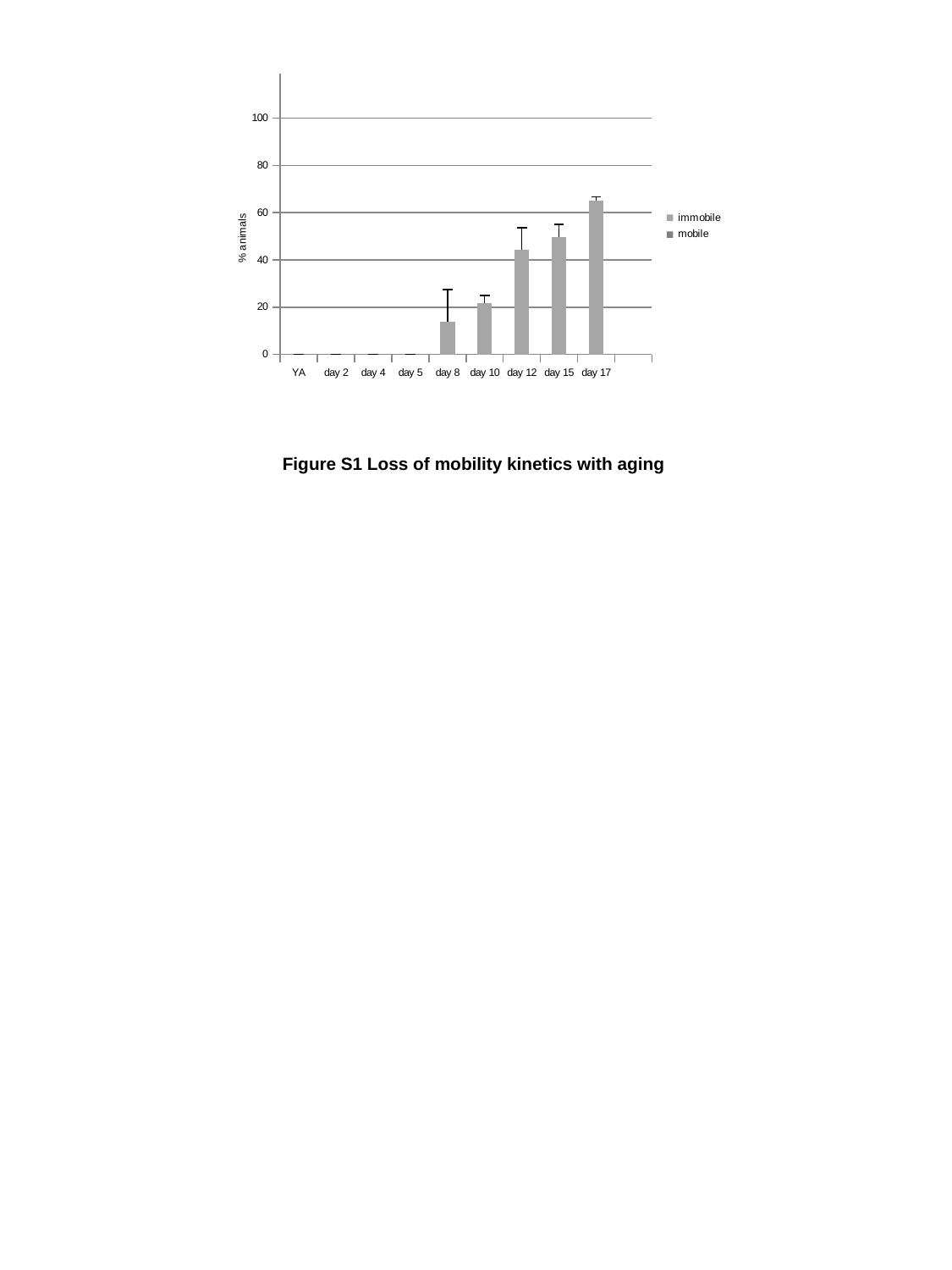

### Chart
| Category | mobile | immobile |
|---|---|---|
| YA | 100.0 | 0.0 |
| day 2 | 100.0 | 0.0 |
| day 4 | 100.0 | 0.0 |
| day 5 | 100.0 | 0.0 |
| day 8 | 86.25 | 13.75 |
| day 10 | 78.12499999999999 | 21.875 |
| day 12 | 55.71428571428572 | 44.28571428571428 |
| day 15 | 50.27777777777778 | 49.72222222222222 |
| day 17 | 34.84848484848485 | 65.15151515151516 |% animals
Figure S1 Loss of mobility kinetics with aging
