# Supplementary material for: UNC‐120/SRF independently controls muscle aging and lifespan in Caenorhabditis elegans
Source: Aging Cell. 2018 Jan 3;17(2):e12713. doi: 10.1111/acel.12713 (PMC5847867; doi:10.1111/acel.12713)
Supplement: Supplementary file 2 [file ACEL-17-e12713-s002.pptx]

## Slide 1
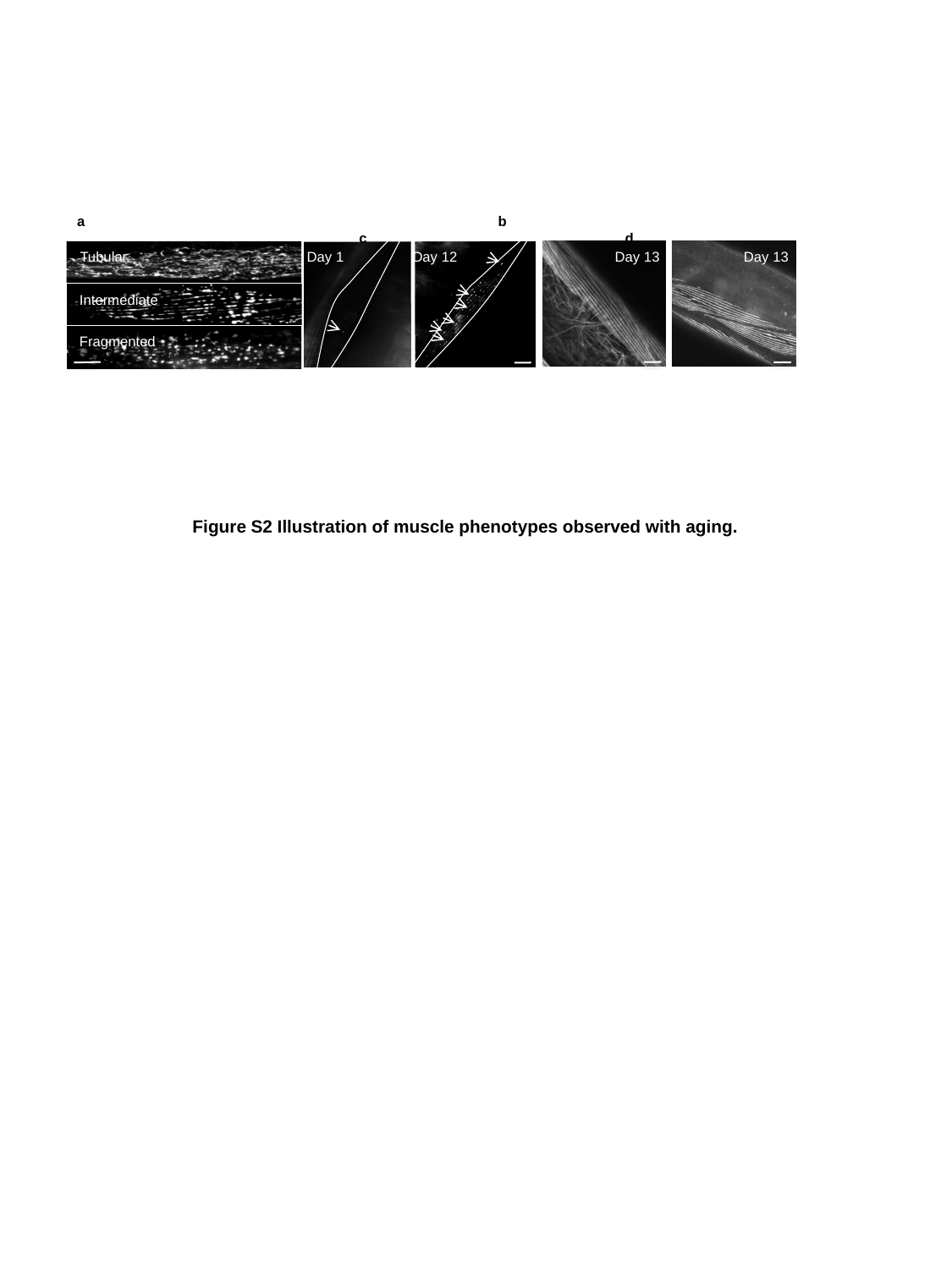

a		 	 b				 c		 d
Day 12
Day 1
Day 13
Day 13
Tubular
Intermediate
Fragmented
Figure S2 Illustration of muscle phenotypes observed with aging.
