# Supplementary material for: UNC‐120/SRF independently controls muscle aging and lifespan in Caenorhabditis elegans
Source: Aging Cell. 2018 Jan 3;17(2):e12713. doi: 10.1111/acel.12713 (PMC5847867; doi:10.1111/acel.12713)
Supplement: Supplementary file 3 [file ACEL-17-e12713-s003.pptx]

## Slide 1
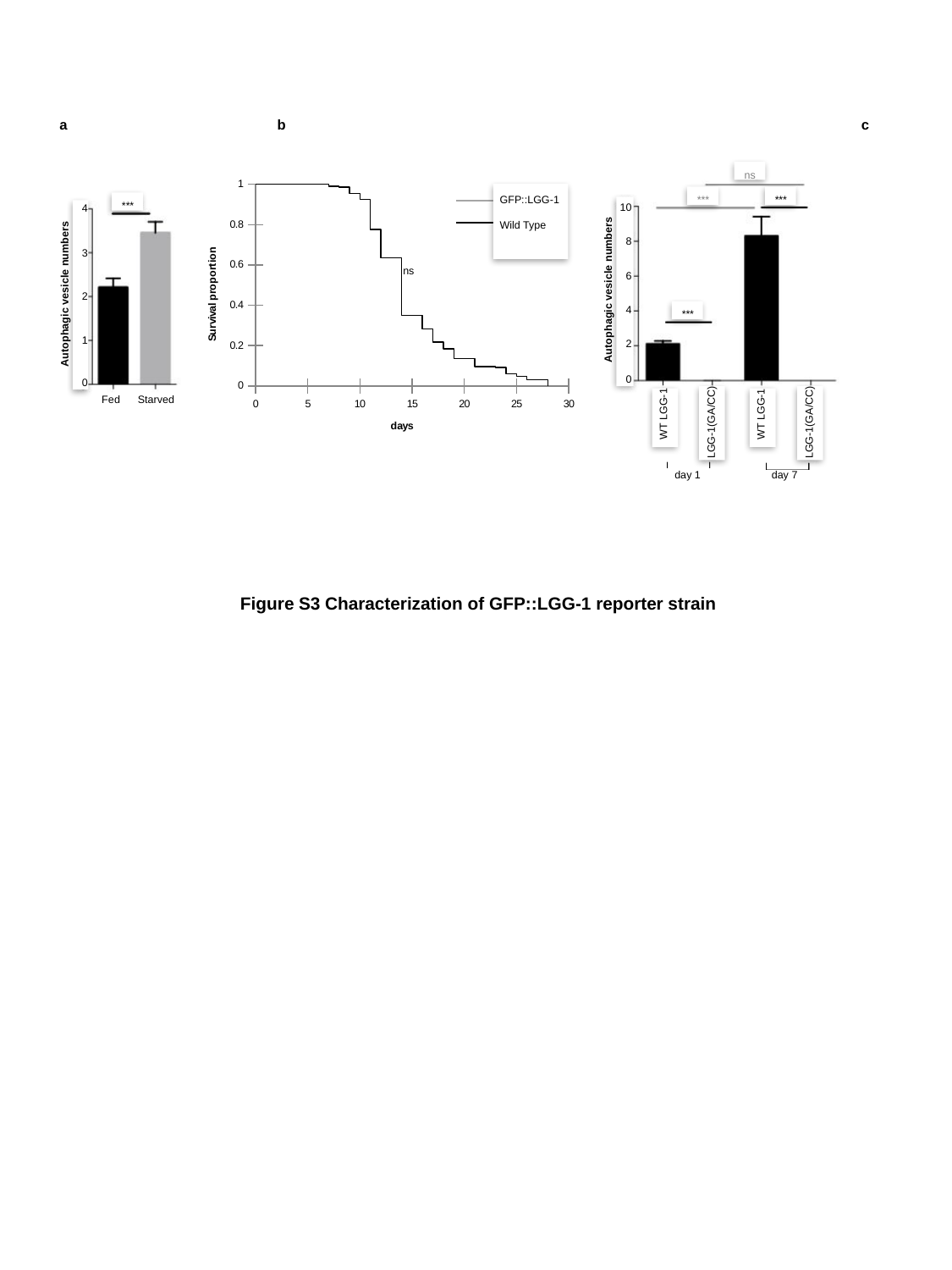

a		 b		 	 	 c
Autophagic vesicle numbers
day 1 day 7
ns
***
***
10
8
6
4
2
0
***
WT LGG-1
WT LGG-1
LGG-1(GA/CC)
LGG-1(GA/CC)
Autophagic vesicle numbers
Fed Starved
***
4
3
2
1
0
### Chart
| Category | | |
|---|---|---|
GFP::LGG-1
Wild Type
ns
Figure S3 Characterization of GFP::LGG-1 reporter strain
