# Supplementary material for: UNC‐120/SRF independently controls muscle aging and lifespan in Caenorhabditis elegans
Source: Aging Cell. 2018 Jan 3;17(2):e12713. doi: 10.1111/acel.12713 (PMC5847867; doi:10.1111/acel.12713)
Supplement: Supplementary file 4 [file ACEL-17-e12713-s004.pptx]

## Slide 1
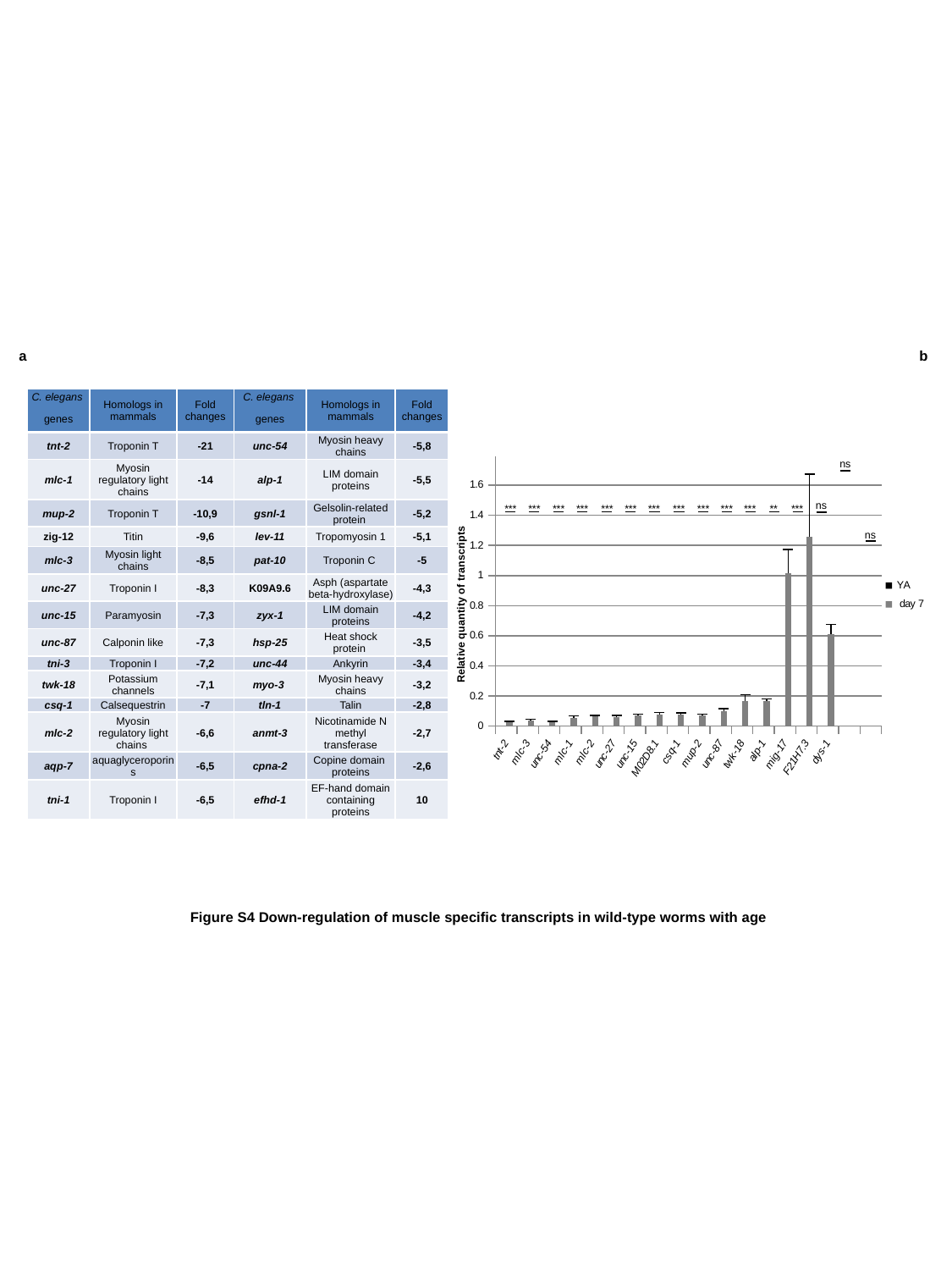

a 		 		 			 b
| C. elegans | Homologs in mammals | Fold changes | C. elegans | Homologs in mammals | Fold changes |
| --- | --- | --- | --- | --- | --- |
| genes | | | genes | | |
| tnt-2 | Troponin T | -21 | unc-54 | Myosin heavy chains | -5,8 |
| mlc-1 | Myosin regulatory light chains | -14 | alp-1 | LIM domain proteins | -5,5 |
| mup-2 | Troponin T | -10,9 | gsnl-1 | Gelsolin-related protein | -5,2 |
| zig-12 | Titin | -9,6 | lev-11 | Tropomyosin 1 | -5,1 |
| mlc-3 | Myosin light chains | -8,5 | pat-10 | Troponin C | -5 |
| unc-27 | Troponin I | -8,3 | K09A9.6 | Asph (aspartate beta-hydroxylase) | -4,3 |
| unc-15 | Paramyosin | -7,3 | zyx-1 | LIM domain proteins | -4,2 |
| unc-87 | Calponin like | -7,3 | hsp-25 | Heat shock protein | -3,5 |
| tni-3 | Troponin I | -7,2 | unc-44 | Ankyrin | -3,4 |
| twk-18 | Potassium channels | -7,1 | myo-3 | Myosin heavy chains | -3,2 |
| csq-1 | Calsequestrin | -7 | tln-1 | Talin | -2,8 |
| mlc-2 | Myosin regulatory light chains | -6,6 | anmt-3 | Nicotinamide N methyl transferase | -2,7 |
| aqp-7 | aquaglyceroporins | -6,5 | cpna-2 | Copine domain proteins | -2,6 |
| tni-1 | Troponin I | -6,5 | efhd-1 | EF-hand domain containing proteins | 10 |
### Chart
| Category | YA | day 7 |
|---|---|---|
| tnt-2 | 1.0 | 0.0264172176559032 |
| mlc-3 | 1.0 | 0.0390216319642067 |
| unc-54 | 1.0 | 0.0284854423942373 |
| mlc-1 | 1.0 | 0.0556536227456566 |
| mlc-2 | 1.0 | 0.0625646085724227 |
| unc-27 | 1.0 | 0.060446587773931 |
| unc-15 | 1.0 | 0.0702020147551823 |
| M02D8.1 | 1.0 | 0.0785158256957674 |
| csq-1 | 1.0 | 0.0742242860267812 |
| mup-2 | 1.0 | 0.0693225350738385 |
| unc-87 | 1.0 | 0.0992566035746296 |
| twk-18 | 1.0 | 0.169000407348414 |
| alp-1 | 1.0 | 0.167000502564236 |
| mig-17 | 1.0 | 1.01524011480958 |
| F21H7.3 | 1.0 | 1.260030671564606 |
| dys-1 | 1.0 | 0.610802719391256 |ns
ns
***
***
***
**
***
***
***
***
***
***
***
***
***
Relative quantity of transcripts
ns
Figure S4 Down-regulation of muscle specific transcripts in wild-type worms with age
