# Supplementary material for: UNC‐120/SRF independently controls muscle aging and lifespan in Caenorhabditis elegans
Source: Aging Cell. 2018 Jan 3;17(2):e12713. doi: 10.1111/acel.12713 (PMC5847867; doi:10.1111/acel.12713)
Supplement: Supplementary file 5 [file ACEL-17-e12713-s005.pptx]

## Slide 1
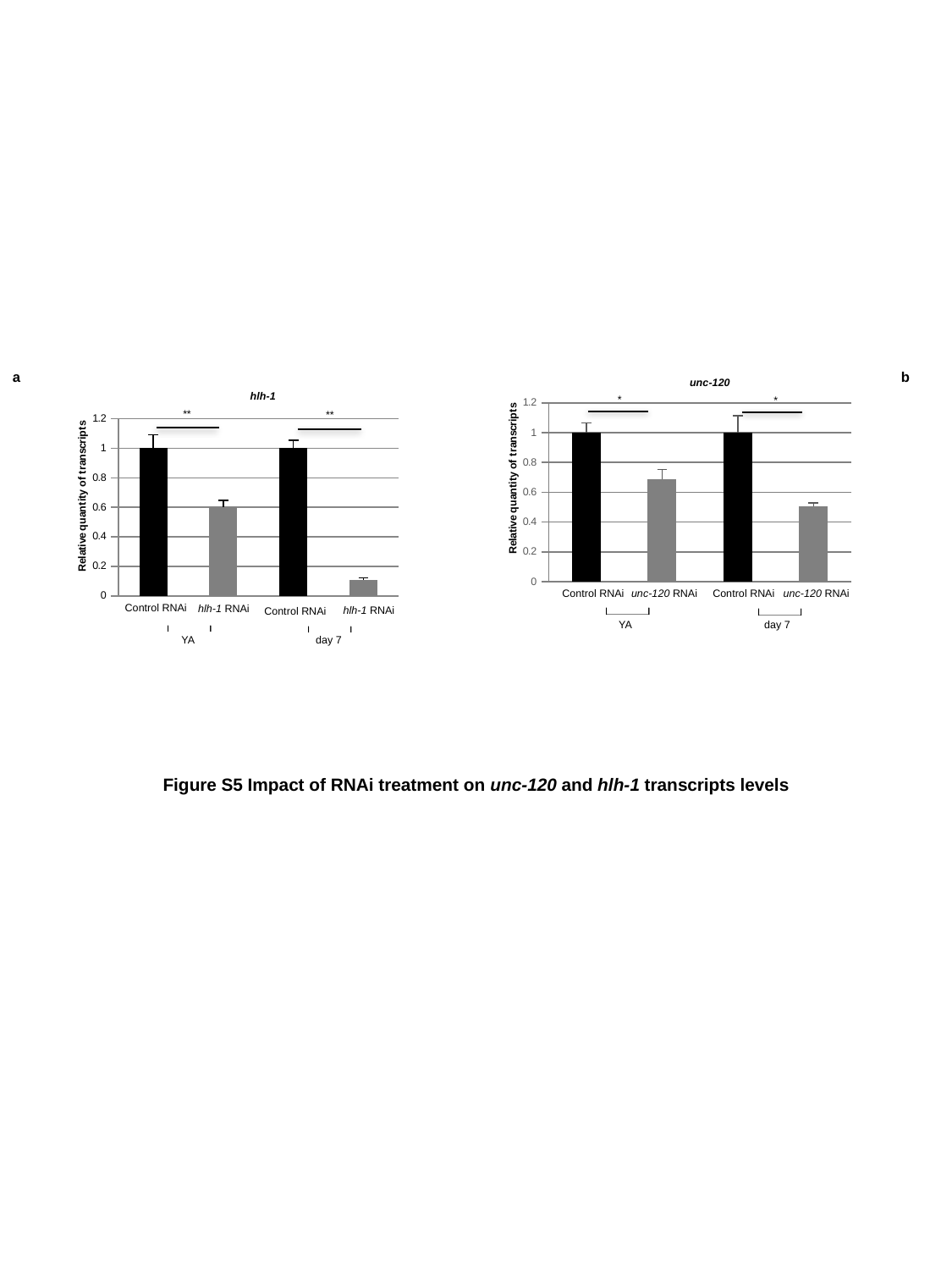

### Chart
| Category | mean |
|---|---|
| Control RNAi | 1.000000003647353 |
| unc-120 RNAi | 0.686151388411798 |
| Control RNAi | 0.999999999566821 |
| unc-120 RNAi | 0.508970940404503 |unc-120
*
*
Control RNAi
unc-120 RNAi
Control RNAi
unc-120 RNAi
YA
day 7
a							b
### Chart
| Category | |
|---|---|
| YA HT115 | 0.999999525742492 |
| YA ARNi hlh-1 | 0.601411844487871 |
| day 7 HT115 | 1.0 |
| day 7 ARNi hlh-1 | 0.109226282950212 |hlh-1
**
**
Control RNAi
hlh-1 RNAi
hlh-1 RNAi
Control RNAi
YA
day 7
Figure S5 Impact of RNAi treatment on unc-120 and hlh-1 transcripts levels
