# Supplementary material for: UNC‐120/SRF independently controls muscle aging and lifespan in Caenorhabditis elegans
Source: Aging Cell. 2018 Jan 3;17(2):e12713. doi: 10.1111/acel.12713 (PMC5847867; doi:10.1111/acel.12713)
Supplement: Supplementary file 6 [file ACEL-17-e12713-s006.pptx]

## Slide 1
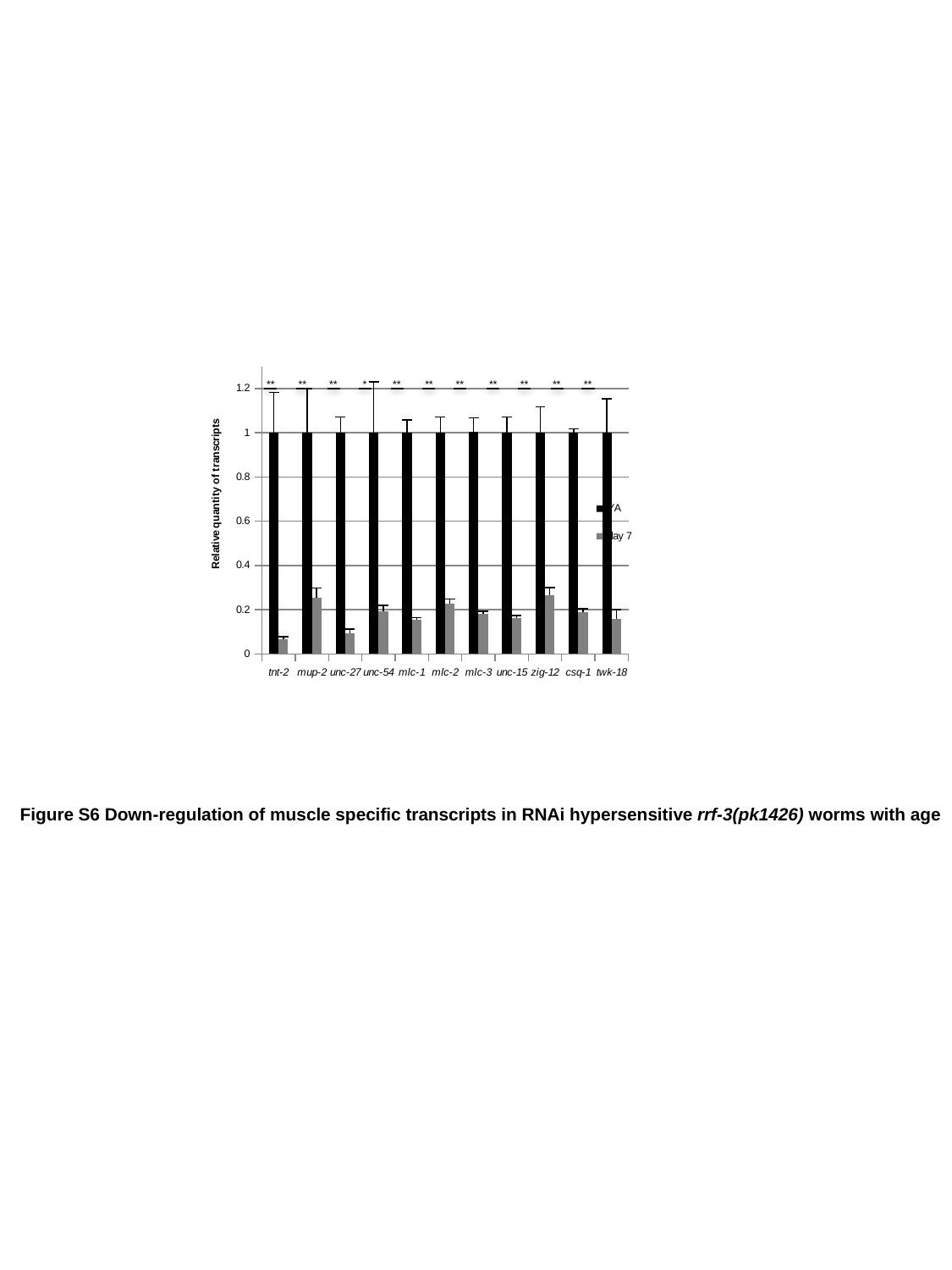

### Chart
| Category | YA | day 7 |
|---|---|---|
| tnt-2 | 1.000000151310656 | 0.0681022198087395 |
| mup-2 | 1.000000014996522 | 0.255466219061508 |
| unc-27 | 0.999999948971361 | 0.092614229112138 |
| unc-54 | 1.000000012125389 | 0.191937681360211 |
| mlc-1 | 1.00000026588394 | 0.153219134952191 |
| mlc-2 | 0.999999777842447 | 0.227118968034465 |
| mlc-3 | 1.004638136953018 | 0.179935012503714 |
| unc-15 | 1.000000254968578 | 0.163495637465672 |
| zig-12 | 0.999999811289966 | 0.267053968604064 |
| csq-1 | 0.999999777311963 | 0.187333968419079 |
| twk-18 | 1.00000007738395 | 0.158074964857644 |**
**
**
**
**
**
**
*
**
**
**
Figure S6 Down-regulation of muscle specific transcripts in RNAi hypersensitive rrf-3(pk1426) worms with age
