# Supplementary figures and images for: UNC‐120/SRF independently controls muscle aging and lifespan in Caenorhabditis elegans
Source: Aging Cell. 2018 Jan 3;17(2):e12713. doi: 10.1111/acel.12713 (PMC5847867; doi:10.1111/acel.12713)

## Slide 1
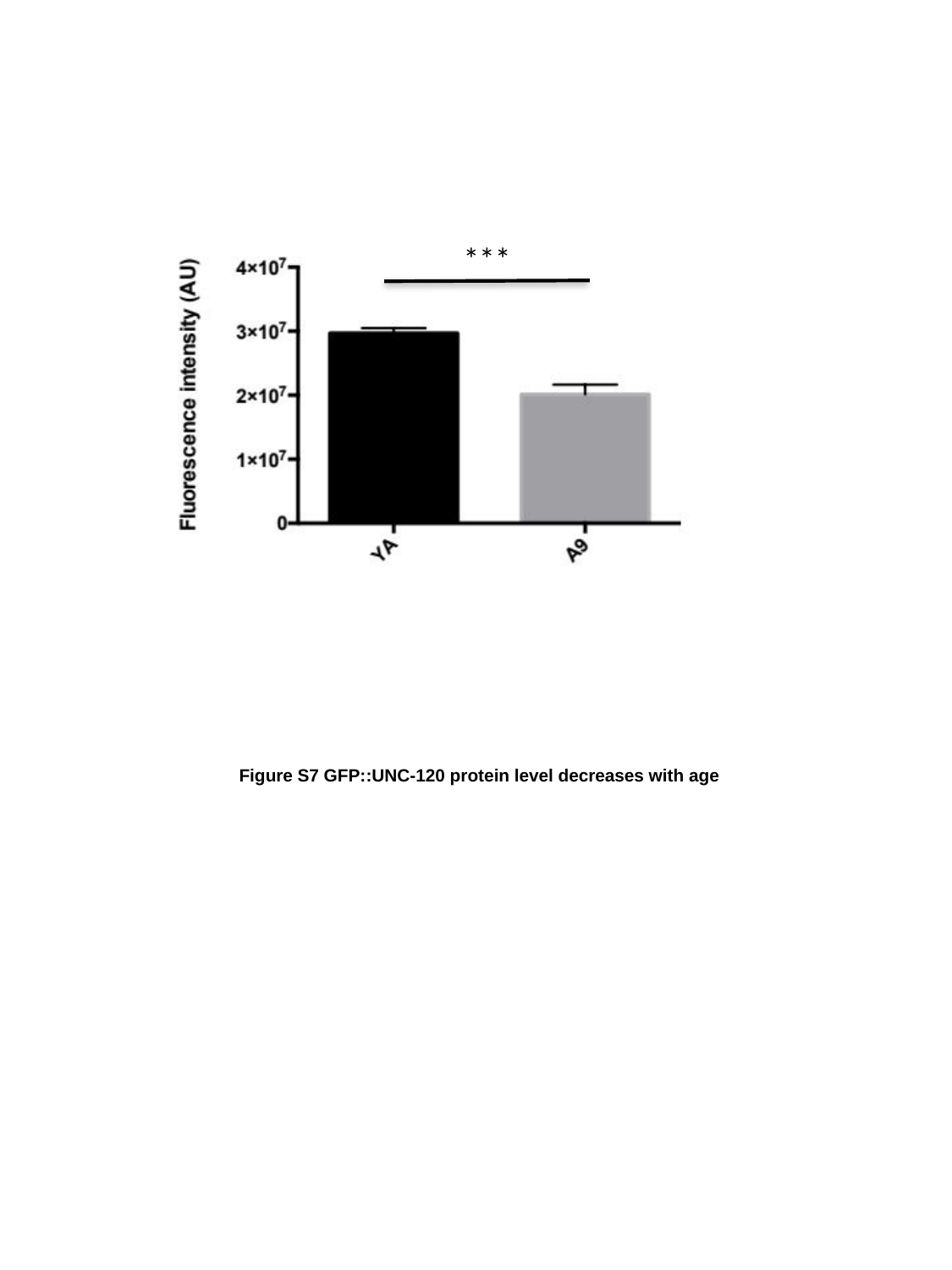

***
Figure S7 GFP::UNC-120 protein level decreases with age

Supplement: Supplementary file 7 [file ACEL-17-e12713-s007.pptx]
