# Supplementary material for: UNC‐120/SRF independently controls muscle aging and lifespan in Caenorhabditis elegans
Source: Aging Cell. 2018 Jan 3;17(2):e12713. doi: 10.1111/acel.12713 (PMC5847867; doi:10.1111/acel.12713)
Supplement: Supplementary file 8 [file ACEL-17-e12713-s008.pptx]

## Slide 1
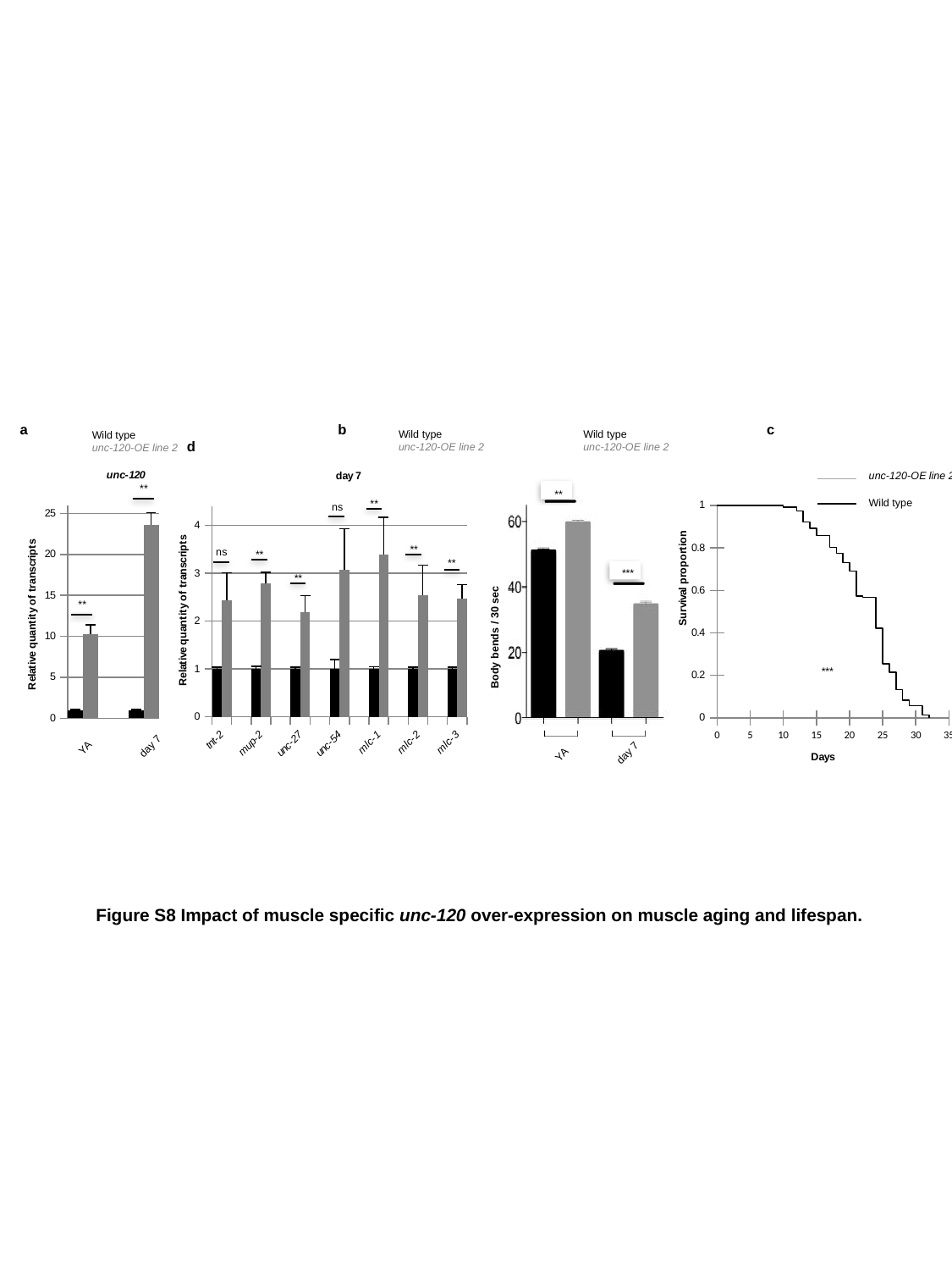

### Chart: unc-120
| Category | N2 | FS439 |
|---|---|---|
| young adults | 1.0 | 10.23894382717127 |
| | None | None |
| 7-day-old adults | 1.0 | 23.58524430642266 |**
**
day 7
YA
Wild type
unc-120-OE line 2
a 		 b			 c			 d
Wild type
unc-120-OE line 2
Body bends / 30 sec
**
***
day 7
YA
Wild type
unc-120-OE line 2
### Chart: day 7
| Category | N2 | FS439 |
|---|---|---|
| tnt-2 | 1.0 | 2.446469620743902 |
| | None | None |
| mup-2 | 1.0 | 2.796635861476233 |
| | None | None |
| unc-27 | 1.0 | 2.185111778723194 |
| | None | None |
| unc-54 | 1.0 | 3.070958977255902 |
| | None | None |
| mlc-1 | 1.0 | 3.386165663339737 |
| | None | None |
| mlc-2 | 1.0 | 2.545493476269228 |
| | None | None |
| mlc-3 | 1.0 | 2.467840376221219 |**
ns
**
ns
**
**
**
### Chart
| Category | | |
|---|---|---|***
unc-120-OE line 2
Wild type
Figure S8 Impact of muscle specific unc-120 over-expression on muscle aging and lifespan.
