# Supplementary material for: UNC‐120/SRF independently controls muscle aging and lifespan in Caenorhabditis elegans
Source: Aging Cell. 2018 Jan 3;17(2):e12713. doi: 10.1111/acel.12713 (PMC5847867; doi:10.1111/acel.12713)
Supplement: Supplementary file 9 [file ACEL-17-e12713-s009.pptx]

## Slide 1
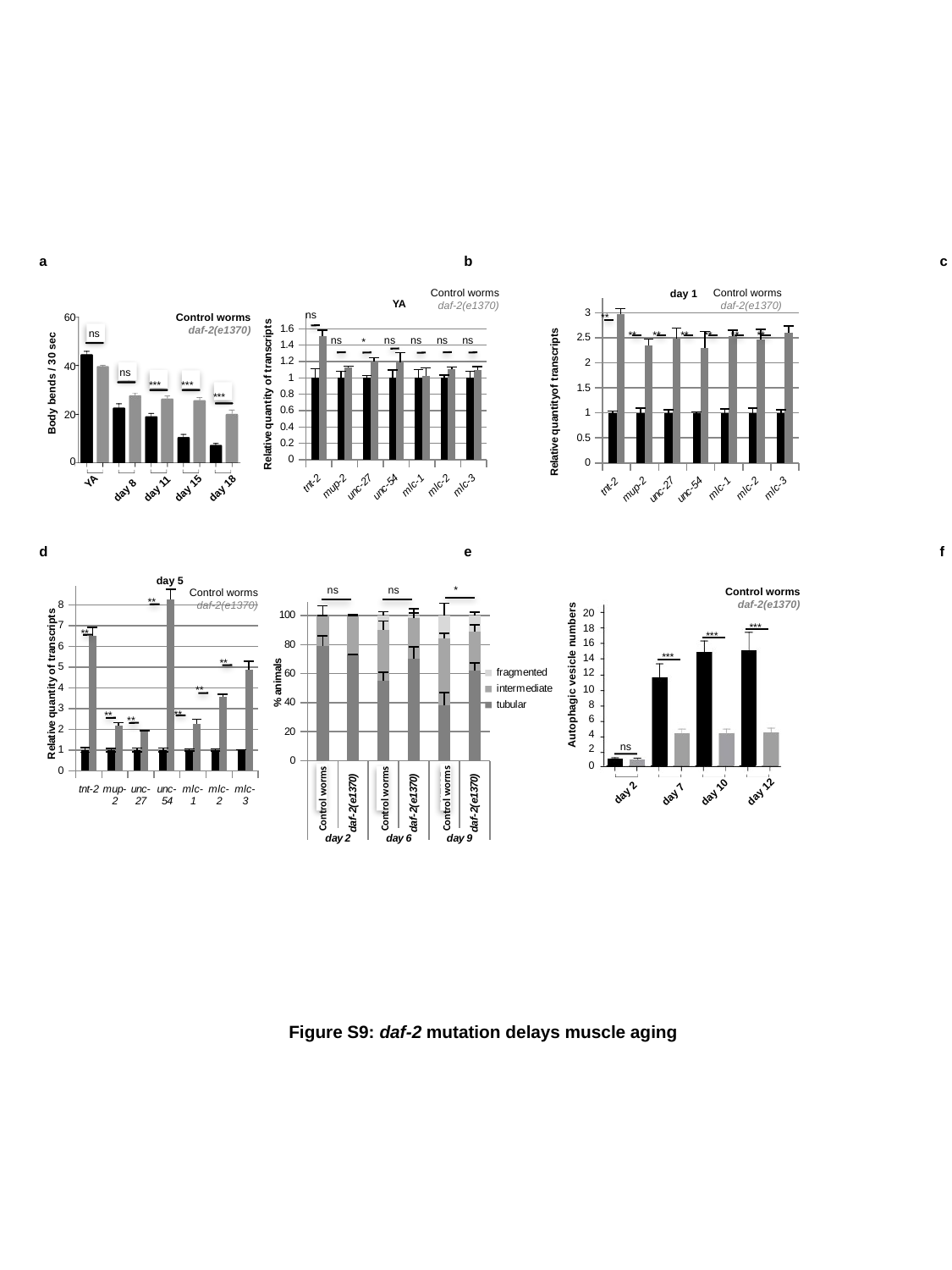

a 		 	 b			 	 c
Control worms
daf-2(e1370)
YA
### Chart
| Category | rrf-3 | rrf-3 daf-2 |
|---|---|---|
| tnt-2 | 0.999999999527227 | 1.510541156606766 |
| mup-2 | 0.999999999482766 | 1.124520036104287 |
| unc-27 | 1.000000558569814 | 1.204314317598751 |
| unc-54 | 0.99999999998417 | 1.186568222920415 |
| mlc-1 | 0.999958503987926 | 1.024596235556671 |
| mlc-2 | 0.999999786959458 | 1.105924539573535 |
| mlc-3 | 1.000000252753838 | 1.100667612085195 |
ns
ns
ns
ns
ns
*
ns
Control worms
daf-2(e1370)
day 1
### Chart
| Category | Jour 1 rrf-3 | Jour 1 rrf-3 daf-2 |
|---|---|---|
| tnt-2 | 1.0 | 2.973737129177928 |
| mup-2 | 1.0 | 2.34605754613441 |
| unc-27 | 1.0 | 2.484827751545348 |
| unc-54 | 1.0 | 2.302501556164182 |
| mlc-1 | 1.0 | 2.52989586684449 |
| mlc-2 | 1.0 | 2.474660311341613 |
| mlc-3 | 1.0 | 2.604409357371724 |**
**
**
**
**
**
**
60
40
20
0
Control worms
daf-2(e1370)
Body bends / 30 sec
ns
ns
***
***
***
YA
day 18
day 11
day 8
day 15
d 		 	 e			 	 f
Control worms
daf-2(e1370)
20
18
16
14
12
10
8
6
4
2
0
***
***
***
Autophagic vesicle numbers
ns
day 2
day 12
day 10
day 7
### Chart
| Category | tubular | intermediate | fragmented |
|---|---|---|---|
| HT115 | 79.16666666666667 | 20.83333333333328 | 0.0 |
| daf-2(e1370) | 72.9166666666667 | 27.08333333333258 | 0.0 |
| HT115 | 54.90196078431372 | 35.29411764705886 | 9.803921568627452 |
| daf-2(e1370) | 70.0 | 28.0 | 2.0 |
| HT115 | 38.0 | 46.0 | 16.0 |
| daf-2(e1370) | 61.81818181818182 | 27.27272727272623 | 10.90909090909091 |ns
ns
*
Control worms
Control worms
Control worms
day 5
### Chart
| Category | Jour 5 rrf-3 | Jour 5 rrf-3 daf-2 |
|---|---|---|
| tnt-2 | 1.0 | 6.50726144442384 |
| mup-2 | 1.0 | 2.18103234219672 |
| unc-27 | 1.0 | 1.886690498713773 |
| unc-54 | 1.0 | 8.266385981139205 |
| mlc-1 | 1.0 | 2.265944304444843 |
| mlc-2 | 1.0 | 3.560892902181229 |
| mlc-3 | 1.0 | 4.859552422258421 |**
**
**
**
**
**
**
Control worms
daf-2(e1370)
Figure S9: daf-2 mutation delays muscle aging
