# Supplementary material for: UNC‐120/SRF independently controls muscle aging and lifespan in Caenorhabditis elegans
Source: Aging Cell. 2018 Jan 3;17(2):e12713. doi: 10.1111/acel.12713 (PMC5847867; doi:10.1111/acel.12713)
Supplement: Supplementary file 10 [file ACEL-17-e12713-s010.docx]

**Supplemental experimental procedures**

***C. elegans* Strains** . Transgenic lines created for this work are described in Table S1. Expression constructs, transgenic animals and generation of knock-in worms are described in [below](http://www.ncbi.nlm.nih.gov/pmc/articles/PMC2034668/#S1).

**Constructs and transgenesis**

Sequences of primers used for transgenes constructions are shown in Table S2. To build the plasmid pKG169: *Pdyc-1S::gfp::lgg-1,* the muscle specific *Pdyc-1S* promoter was PCR amplified from the *dyc-1::gfp* plasmid (Lecroisey *et al.* 2008) using the CG40 and CG41 primers and inserted into the KpnI/SphI sites of the *pGFP::lgg-1* plasmid (gifted by Alicia Meléndez (Melendez *et al.* 2003)) to replace the *lgg-1* promoter by the *dyc-1S* promoter. pKG169 was injected at 5ng/μL with the *Pmyo‑2::mCherry* (expressed in pharyngeal muscle) plasmid pCFJ90 as a co-injection marker (2,5ng/μl)) and the pBSC plasmid as a DNA carrier (100ng/μl, Addgene, Cambridge, MA) into N2 worms. *kagIs1[Pdyc-1S::GFP::lgg-1]* was obtained after integration of the corresponding extrachromosomal array by UV (Mariol *et al.* 2013) and backcrossed 2 times to obtain the strain KAG238 used in this study. To build the pKG185 *[Pdyc-1S::gfp::lgg-1GA>CC]* plasmid the same strategy was used starting from plasmid *Plgg-1::gfp::lgg-1(G116A)* kindly gifted by Renaud Legouis (Manil-Segalen *et al.* 2014). This plasmid was injected and integrated in the genome of N2 worms as described above. The KAG350 used in this study was backcrossed 2 times with N2.

To generate the pFS47 : *Pmyo-3 ::gfp::unc-120* plasmid, *unc-120* and *gfp* were PCR amplified from the fosmid clone WRM0611bD01 using primers oLM29 and oLM35, and from pMP18 using primers oLM33 and oLM34, respectively. Both fragments were cloned into pPD95.86 (Addgene, Cambridge, MA) by Gibson assembly. The resulting construct pFS47, together with the *Pmyo-2::mCherry* co-marker, were microinjected into N2 worms. Integration of the extrachromosomal arrays was achieved by X-ray irradiation (30 Gys) of transgenic worms (X-RAD 320 chamber, from PXI Precision X ray). Four independent lines were obtained, outcrossed at least three times to N2 worms and showed the same expression pattern. FS438, (*fsIs438[Pmyo-3 ::gfp::unc-120*]) and FS439 (*fsIs439[Pmyo-3 ::gfp::unc-120*]) were further characterized and referred as *unc-120* OE line 1 and *unc-120* OE line 2 in the text.

GFP tagging of the endogenous *unc-120* locus (*fsIs442[gfp::unc-120, I:8460225])* was performed by CRISPR with two gRNAs (GTGAGCAAAGTCTTCGGCTT and GAGCAGTTGAGCAAAGTCTT) that target the 5' end of *unc-120* coding region. gRNAs were cloned into PmeI/SexA1 digested pPT02 (kind gift from Thomas Boulin) by Gibson assembly using oLM53/oLM54 and oLM55/oLM56 primers. The pPT02 plasmid allows expression of guide fused with sgRNA scaffold under the control of the U6 pol III promoter. To generate the *unc-120* repair plasmid *unc-120* homologous regions and GFP coding region were obtained by PCR amplification from the fosmid clone WRM0611bD01 using primers oLM47/oLM48 and oLM51/oLM52, or from plasmid pMP18 using primers oLM49/oLM50, respectively. *unc-120* homologous regions and GFP coding PCR products were cloned into EcoRV/PstI digested pBluescript by Gibson assembly. *unc-120(st364)* mutant worms were injected with a mix containing the repair template (50 ng/ µl), gRNAs (50 ng/ µl), pDD162 Cas9 expressing plasmid (6) (50 ng/μl) and pCFJ90 (*Pmyo-2::mCherry)* co-marker (2,5 ng/ µl). As *unc-120(st364)* mutant worms are paralyzed at 25°, F2 progeny was shifted to this restrictive temperature and transgenic worms were identified based on their wild-type mobility phenotype.

SL2::tagRFP-T tagging of the endogenous *tnt-2* locus (cf Figure 3) was performed by CRISPR with two gRNAs targeting the 3' end of *tnt-2* coding region CGTTTAGGCTGGAACGGCCT and GACGGTTGGAGCGTTTAGGC. gRNA were cloned into PmeI/SexA1 digested pPT02 plasmid by GIBSON assembly, using oAM40/oAM41 and oAM42/oAM43 oligos. The repair template containing SL2 and tagRFP-T DNA, flanked by 30 bp of *tnt-2* homologous regions, was obtained by PCR amplification of *SL2 ::tagRFP-T* fragment from pAM019 by using oAM44 and oAM45 oligos. The two *gRNA* and the repair template were co-injected into wild-type worms, together with pDD162 Cas9 expressing plasmid, a third guide targeting the *dpy-10* locus and a second repair template for *dpy-10* which introduced the *cn64* mutation (7). F2 transgenic worms were isolated based on the Dumpy phenotype and on the expression of the fluorescent tagRFP-T and then backcrossed with N2 in order to remove *dpy-10(cn64)* mutation and to obtain FS410 (*fsIs410[tnt-2::SL2::tagrfp-t, X:8723552]).*

GFP tagging of the endogenous *myo-3* locus was performed by CRISPR with a guide RNA targeting the 5' end of *myo-3* coding region (GGTATTGCAAAGAGTTATGA). The guide sequence was inserted in the NarI/SphI site of plasmid pDD162 by GIBSON assembly. The repair template containing GFP sequence flanked by 800 and 1300 bp long homologous regions of *myo-3* (containing mutated PAMs to prevent Cas9 from cleaving the repair template) was amplified by PCR and inserted in the SmaI site of pBluescript site by GIBSON assembly. The *gRNA* containing plasmid (pKG202) and the repair template (pKG204) were co-injected, into wild type worms. In the progeny worms carrying an insertion of the GFP coding sequence in the *myo-3* gene were identified by PCR. The resulting strain was named KAG420 *kagIs4[gfp::myo-3]*.

**Processing of transgenic strains for mitochondria, autophagic vesicles and myofilaments analysis**

Fluorescent transgenic worms were fixed in 1 mL of PBS 1X supplemented with 13 µl of 37% formaldehyde for 30 minutes except for the *gfp::lgg-1* strains that were fixed 20 minutes. For the actin staining, N2 worms were then stained according to (Waterston *et al.* 1984). Quantification of mitochondrial morphology in body wall muscle cells was performed in double blind according to the following criteria, (1) cells containing long interconnected mitochondrial networks were classified as tubular; (2) cells containing a combination of interconnected mitochondrial networks along with some smaller fragmented mitochondria were classified as intermediate; (3) cells with sparse small round mitochondria were classified as fragmented. For each worm one muscle quadrant (corresponding to 23 to 24 cells) was observed. Although some variations existed between muscle cells, we always observed a predominant mitochondria pattern that correspond to 70 to 100% of muscle cells observed and which was used to classify the worm. Comparisons between different conditions were performed thanks to Chi2 test by pooling intermediate and fragmented classes when the number of worms counted in one class was less than 5. The number of autophagic vesicles was also determined in blind from the observation of one muscle quadrant per worm. For starvation experiments, gravid adult worms (strain KAG238) were let to lay eggs for 8 hours at 15°C. After 4 days of culture at 15°C, L4 worms were transferred on plates containing OP50 bacteria (fed) or not (starved) for 24 hours at 15°C. Plates were supplemented with antibiotics to avoid bacteria growth (ampicillin (100µg/ml) and tetracycline (12,5µg/ml). For GFP-LGG-1 punctea quantification, worms were collected and fixed as indicated above.

All observations were done at 20°C, except for starvation experiments. Comparisons were performed thanks to Kruskal-Wallis and Dunn’s test using the PRISM6.0 software.

**Microscopy and image analysis**

For mitochondria, autophagic vesicles and actin observations worms were mounted in DAKO fluorescence medium and scored using a Zeiss AxioImager, Z1 upright, fitted with a 63 or 100x objective. For Fig 1A and Fig 3 and for KAG420 worms were mounted on agarose pad in M9 solution containing 2% polystyrene beads (Polysciences, 0.1 µm diameter) and observed thanks to an inverted confocal microscope (Olympus IX83) equipped with a CSU spinning disk (Yogokawa) and an EMCCD camera (iXon ultra 888) driven by Andor IQ3.3. Projected z-stack images were obtained using Fidji software.

For GFP::UNC-120 quantification, a 100μm region anterior to the vulva was imaged (with1μm Z step). After sum intensity projection, the total fluorescence in 3 to 6 nuclei per worm were quantified using ROI and measurement tools of Fidji software. For each nucleus, the fluorescence background was measured in its vicinity and the value subtracted from the specific intensity.

**RNAi feeding, lifespan assay**

In all RNAi expreiments *rrf-3(pk1426*) mutants worms were used in order to increase RNAi sensitivity (Simmer *et al.* 2002). Worms were maintained on OP50 plates and transferred on RNAi plates at the L4 stage. The control (HT115), D1081.2 (*unc-120*) and B0304.1 (*hlh-1*) clones were purchased from GeneService. All clone were verified by sequencing*.* In all RNAi experiments *rrf-3(pk1426)* mutants were used in order to improve RNAi efficiency as previously described (Simmer *et al.* 2002).

**Identification of muscle specific genes whose expression vary with lifespan expectancy**

In order to identify genes whose expression vary with lifespan expectancy we compared the transcriptome of young-adult (YA) and 7-day-old worms (A7) of different genetic background (*wild-type,* short lived *daf-18(e1375),* long lived *slcf-1(tm2258)* and *daf-18(e1375); slcf-1(tm2258) (Mouchiroud et al. 2011) ).* To this end total RNAs were isolated from 3 independent biological replicates. For each sample 15 μg of ARNc were hybridized to an Affymetrix *C. elegans* Genome Arrays after a single round of amplification from 100 ng of total RNA (Kit GeneChip 3’ IVT Express, Affymetrix). The obtained data were normalized with Affymetrix Expression Console software using MAS5 statistical algorithm. Normalized data were compared and filtered using Partek Genomic Suite software 6.6 (Partek Inc., St. Louis, MO, US). A gene was considered as differentially expressed when a comparison between two groups yielded a P-value < 0.05 in the student parametric test and if a more than 2-fold variation was observed. PCA and hierarchical clustering based on the differentially expressed genes were performed to evaluate the relationships between different genotypes and ages. Strikingly, the 24 samples were separated into clusters that reflected worm physiological age: all YA from all genotypes were grouped as one cluster while 7-day-old worms samples were distributed along the PC1 axis with samples from longest living worms being nearest the YA cluster, while samples from the shortest living being farthest from the YA cluster. PC2 that reflected differences between the three replicates per condition accounted for 10% of variations, while PC1 account for 23.3% of the variations. The list of genes mentioned in Table S1 referred to muscle specific genes (according to Wormbase) identified among the list of genes that explain PC1. Full data sets are available upon request.

**Reverse transcription– qPCR**

Wild-type worms (Fig 2CD, 4AB, S4B, S8AB) or *rrf-3(pk1426) (*Fig.1E, 2AB, 5E, S5AB, S6, S9B-D) mutants worms were collected at different ages and stored at -80°C in 1mL TRIzol (Invitrogen), after flash-freezing in liquid N2. RNAs were extracted using the phase separation followed by phenol−chloroform purification. Total RNA were quantified using DO 260 nm on a NanoDrop 1000 spectrophotometer (ThermoScientific) and their quality was assessed by RIN measurement (RNA integrity number) using a Agilent 2100 Bioanalyzer. DNase treatment was performed with the TurboDNA-free kit (Thermo Fisher Scientific) and cDNA was then synthesized with the iScript cDNA synthesis kit (Bio-Rad) following provider's instructions. qPCR reactions were performed using iTaq Universal SYBR (Biorad) in a CFX96 Realtime System C1000 Thermal Cycler (Bio-Rad). The experimental protocol consisted of an initial step at 95°C for 4 min, followed by an amplification program during 40 cycles (30 sec at 95°C, 30 sec at 60°C and 30 sec at 72°C). Melting-curve analyses were performed to verify the amplification of a single product. Reference genes *tba-1*, *pmp-3* and Y45F10D.4 proved to be stable between assays and were used for normalization. All primers (reported in Table S3 below) were designed using NCBI Primer - BLAST and selected to generate amplicons with a length of 100−200 bp. Standard curves were generated for each primer set to calculate the efficiency of each set. Only primer sets with an efficiency of 1.8−2.1 were used for RT qPCR. RT qPCR experiments were repeated at least three times using independent biological samples. Comparison were done thanks to Student t test or Mann-Whitney test.

**Statistical analysis**

All the statistical analyses were performed using GraphPad Prism version 6 (GraphPad, La Jolla, CA, USA), excepted for survival analyses, which were performed using XLSTAT software (Addinsoft, NY, USA). Lifespan data were analysed using the Kaplan−Meier method and were compared using the log rank test. For the mitochondria phenotype comparisons between different conditions were performed thanks to Chi2 test by pooling intermediate and fragmented classes when the number of worms counted in one class was less than 5. The Kruskal–Wallis analysis was performed to determine if any of the sample conditions originated from a different distribution than the others. The Kruskal–Wallis analysis allows for differing sample sizes and makes no assumption as to the shape of the originating distribution. A subsequent Dunn’s test for multiple comparisons was performed to determine which sample sets, if any, originated from differing distributions than other sample sets. All test were performed in two-tailed approaches. Compared samples were considered different when statistical test gave a P-value <0.05 (*p<0.05; **p<0.01; ***p<0.001), ns: non significant.

Representative results from at least three independent biological replicates are shown unless stated otherwise.

Table S1. List of generated strains

| Strains | Genotypes |
| --- | --- |
| FS462 | *kagIs1[Pdyc-1S::gfp::lgg-1+ Pmyo‑2::mCherry];rrf-3(pk1426)* |
| FS423 | *daf-2(e1370); rrf-3(pk1426)* |
| FS424 | *daf-2(e1370); zcIs14 [Pmyo-3::GFP(mitochondria)]* |
| FS438 | *fsIs438[Pmyo-3::gfp::unc-120+ Pmyo‑2::mCherry]* |
| FS439 | *fsIs439[Pmyo-3::gfp::unc-120+Pmyo‑2::mCherry],* |
| FS442 | *fsIs442[gfp::unc-120 I:8460225]* |
| FS449 | *fsIs410[tnt-2::SL2::tagrfp-t, X:8723552]; fsIs442[gfp::unc-120 I:8460225]* |
| FS454 | *fsIs438[Pmyo-3 ::gfp::unc-120+Pmyo‑2::mCherry]; zcIs14[Pmyo-3::GFP(mitochondria)]* |
| FS463 | *kagIs1[Pdyc-1S::gfp::lgg-1+Pmyo‑2::mCherry]; fsIs438[Pmyo- ::gfp::unc-120+ Pmyo‑2::mCherry]* |
| FS464 | *daf-2(e1370); rrf-3(pk1426); kagIs1[Pdyc-1S::gfp::lgg-1+Pmyo‑2::mCherry]* |
| FS466 | *daf-2(e1370); kagIs1[Pdyc-1S::gfp::lgg-1+Pmyo‑2::mCherry]* |
| KAG238 | *kagIs1[Pdyc-1S::gfp::lgg-1+Pmyo‑2::mCherry]* |
| KAG350 | *kagIs3[Pdyc-1S::gfp::lgg-1GA>CC+Pmyo‑2::mCherry]* |
| KAG420 | *kagIs4[gfp::myo-3, V:12226816]* |
|  |  |

Table S2. Primers used for transgenes constructions:

| Name | Primer Sequence 5’to 3 |
| --- | --- |
| CG40 | AATTGCATGCCCCCTCGAGGTCGACATTAC |
| CG41 | AATTGGTACCTTGTGAGTAGCGTAGAGTGC |
| oLM29 | GGCCGATGCGGAGCTCTTCATTTGATGCAATGGCTGC |
| oLM33 | CTAGATCCATCTAGAGGATCCATGAGTAAAGGAGAAGAACTTTTC |
| oLM34 | TCGGTCATTTTGTATAGTTCATCCATGCC |
| oLM35 | ACTATACAAAATGACCGAAGCCGAAGAC |
| oLM47 | CTCGAGGTCGACGGTATCGATAAGCTTGATCTTCTTGCTTCTTTTGCC |
| oLM48 | TTCTCCTTTACTCATGTTCTGAAATTTTGGAAGGTG |
| oLM49 | CCAAAATTTCAGAACATGAGTAAAGGAGAAGAACTTTTC |
| oLM50 | TTCAGCTTCAGTCATTTTGTATAGTTCATCCATGCC |
| oLM51 | GATGAACTATACAAAATGACTGAAGCTGAAGACTTTGCTCAACTGCTTC |
| oLM52 | CTCTAGAACTAGTGGATCCCCCGGGCTGCATCGCATTCCGTTTCCACC |
| oLM53 | ATTGCAAATCTAAATGTTTGTGAGCAAAGTCTTCGGCTTGTTTTAGAGCTAGAAATAGCA |
| oLM54 | TGCTATTTCTAGCTCTAAAACAAGCCGAAGACTTTGCTCACAAACATTTAGATTTGCAAT |
| oLM55 | ATTGCAAATCTAAATGTTTGAGCAGTTGAGCAAAGTCTTGTTTTAGAGCTAGAAATAGCA |
| oLM56 | TGCTATTTCTAGCTCTAAAACAAGACTTTGCTCAACTGCTCAAACATTTAGATTTGCAAT |
| oAM040 | AATTGCAAATCTAAATGTTTGCGTTTAGGCTGGAACGGCCTGTTTTAGAGCTAGAAATAG |
| oAM041 | CTATTTCTAGCTCTAAAACAGGCCGTTCCAGCCTAAACGCAAACATTTAGATTTGCAATT |
| oAM042 | AATTGCAAATCTAAATGTTTGACGGTTGGAGCGTTTAGGCGTTTTAGAGCTAGAAATAGC |
| oAM043 | GCTATTTCTAGCTCTAAAACGCCTAAACGCTCCAACCGTCAAACATTTAGATTTGCAATT |
| oAM044 | AGGTTGTGATCCCAGATAGTGAAGCTGCTGCAGAGGCCGTTCCAGCCTAAGCTGTCTCATCCTACTTTCA |
| oAM045 | TGAGGTGGGGTATGATGACGGTTGGAGCGTCTATTTGTATAGTTCATCCATG |

Table S3. Primers used for RT-qPCR :

| mRNA | Primer Sequence 5’to 3 | mRNA | Primer Sequence 5’to 3 |
| --- | --- | --- | --- |
| *tba-1* | AGCGCGCCTTCGTTCACTGG GGAGCAGGCGGCACGAGTTT | *zig-12* | GTCGGTGTTCCTTGAGGTCATC ACGGCTTTGTATTCTCCGGC |
| *pmp-3* | GTTCCCGTGTTCATCACTCAT ACACCGTCGAGAAGCTGTAGA | *csq-1* | GAGCCAGGATCAGTTGCTCTC TCTGCGCATTTGGACCTCTC |
| *Y45F10D*.*4* | TACGAGAACCCGCGAAATGT TTCGGTTGCCAGGGAAGATG | `  *mup-2* | AGGCCTTCATGCTCGTATCG TGACGTTGACGCTCGTGTAA |
| *tnt-2* | AAAGAGCTCAACGAGCGTCA ATCGAACTTGGAGGCTGTGG | `  *twk-18* | TGCGCAAGGAGTTTCTACGAT ACAGTTCGCCGGATGAGTTC |
| *mlc-3* | GGGAGAGATTGACAGGAACTGAC CCTCTCCTCCCTCGATTGGT | *mig-17* | CCAAGACACGAACACGCAGT CATGAAGCGCTCCAAGTGAGT |
| *unc-54* | GAGCGCTCCAAGAAGGCTAT GAGCTGTTGCTCAAGTCCCT | *dys-1* | CGAGGGAGAGGATTACGATGC GGATTCGTATCGTTCGGC |
| *mlc-1* | GCCAGATCGACGCCATGATTAAA ATAATGGTGGCCTCTGGGTCA | *F21H7.3* | CTCATGGAGGATGTTCCGCA GGTCGGCCGAGTTGTTCATA |
| *mlc-2* | CGGAACCCAAGTCGGAGATG AGTCAGCGTAGGTTCCTTGC | *unc-120* | TGCCTTAATGCTCCTGGTGG GGGGCGAGAAATGGAGTGAA |
| *unc-27* | GTGAAGAAGCCGGAGAGGAC CGGCCTTGACCATCAAAAGC | *hlh-1* | TTCAGAGCCGAGTCGACAGT CGCCTTCCGTCTGTCCAATTT |
| *unc-15* | AAGAAGGATCGCCGCATCAA CGTGTTGGTATCTGCGGACT |  |  |

References:

1. Mariol, M. C., Walter, L., Bellemin, S., & Gieseler, K. (2013). A rapid protocol for integrating extrachromosomal arrays with high transmission rate into the *C. elegans* genome. *Journal of Visualized Experiments*: *JoVE*, e50773.
2. Mouchiroud, L., Molin, L., Kasturi, P., Triba, M. N., Dumas, M. E., Wilson, M. C., . . . Solari, F. (2011). Pyruvate imbalance mediates metabolic reprogramming and mimics lifespan extension by dietary restriction in *Caenorhabditis elegans*. *Aging Cell, 10*, 39–54. https://doi.org/10.9 1111/j.1474-9726.2010.00640.x
3. Waterston, R. H., Hirsh, D., & Lane, T. R. (1984). Dominant mutations affecting muscle structure in *Caenorhabditis elegans* that map near the actin gene cluster*. Journal of Molecular Biology, 180*, 473–496. https://doi.org/10.1016/0022-2836(84)90023-8
